# LOXL2-enriched small extracellular vesicles mediate hypoxia-induced premetastatic niche and indicates poor outcome of head and neck cancer

**DOI:** 10.7150/thno.62455

**Published:** 2021-09-03

**Authors:** Guiquan Zhu, Linlin Wang, Wanrong Meng, Shun Lu, Bangrong Cao, Xinhua Liang, Chuanshi He, Yaying Hao, Xueyu Du, Xiaoyi Wang, Longjiang Li, Ling Li

**Affiliations:** 1 Department of Head and Neck Oncology, West China Hospital of Stomatology, State Key Laboratory of Oral Diseases, National Clinical Research Center for Oral Diseases, Sichuan University, Chengdu, 610041; 2 Sichuan Key Laboratory of Radiation Oncology, Sichuan Cancer Hospital & Institute, Sichuan Cancer Center, School of Medicine, University of Electronic Science and Technology of China, Chengdu, 610041

**Keywords:** small extracellular vesicle, premetastatic niche, hypoxia, head and neck squamous cell carcinoma, Lysyl Oxidase Like 2

## Abstract

Small extracellular vesicles (sEVs) operate as a signaling platform due to their ability to carry functional molecular cargos. However, the role of sEVs in hypoxic tumor microenvironment-mediated premetastatic niche formation remains poorly understood.

**Methods**: Protein expression profile of sEVs derived from normoxic and hypoxic head and neck squamous cell carcinoma (HNSCC) cells were determined by Isobaric Tagging Technology for Relative Quantitation. In vitro invasion assay and in vivo colonization were performed to evaluate the role of sEV-delivering proteins.

**Results**: We identified lysyl oxidase like 2 (LOXL2) which had the highest fold increase in hypoxic sEVs compared with normoxic sEVs. Hypoxic cell-derived sEVs delivered high amounts of LOXL2 to non-hypoxic HNSCC cells to elicit epithelial-to-mesenchymal transition (EMT) and induce the invasion of the recipient cancer cells. Moreover, LOXL2-enriched sEVs were incorporated by distant fibroblasts and activate FAK/Src signaling in recipient fibroblasts. Increased production of fibronectin mediated by FAK/Src signaling recruited myeloid-derived suppressor cells to form a premetastatic niche. Serum sEV LOXL2 can reflect a hypoxic and aggressive tumor type and can serve as an alternative to tissue LOXL2 as an independent prognostic factor of overall survival for patients with HNSCC.

**Conclusion**: sEVs derived from the hypoxic tumor microenvironment of HNSCC can drive local invasion of non-hypoxic HNSCC cells and stimulate premetastatic niche formation by delivering LOXL2 to non-hypoxic HNSCC cells and fibroblasts to induce EMT and fibronectin production, respectively.

## Introduction

Head and neck squamous cell carcinoma (HNSCC), including the epithelial neoplasms of the lip, oral cavity, oropharynx, hypopharynx, and larynx, is a serious and growing problem in many parts of the world. The estimated annual incidence is approximately 275,000 worldwide 1. Despite numerous advances in the diagnosis and treatment of HNSCC, the overall five-year survival rates for patients with localized HNSCC are higher than 80% but drop to 40% when local metastases are involved, and further decrease to 20% in patients with advanced disease (stage IV) 2. Metastasis remains the cause of over 90% of cancer-related deaths. The mechanisms mediating this highly aggressive nature of HNSCC remain insufficiently investigated.

The ''seed and soil'' theory, which explains organotropism in metastasis, has contributed to our understanding of tumor metastasis; the theory implies that disseminated tumor cells (''seed'') colonize in specific organ sites (''soil'') where the microenvironment is favorable for metastasis 3. Indeed, the primary tumor can form a microenvironment in distant organs before disseminated cells arrive at the second site 4. This predetermined microenvironment is termed pre-metastatic niche (PMN) 4. The extracellular matrix, with components such as fibronectin (FN) and hyaluronan, constitutes a scaffold that supports the attachment and reactivation of cancer cells in a metastatic site 5. Additionally, the increased recruitment of myeloid-derived suppressor cells (MDSCs) is a fundamental characteristic of PMNs. Recently, the hypoxic tumor microenvironment within the primary tumor was demonstrated to be an important contributor to PMN formation 6.

Hypoxia is one of the most actively studied characteristics of the tumor microenvironment 7. High rates of proliferation of cancer cells result in rapid tumor mass development that exceeds the rate of blood vessel growth, thus forming an avascular environment deficient in oxygen. The mechanical regulation of cancer biology by hypoxia is mainly mediated by the members of hypoxia-inducible factor (HIF) family, specifically HIF-1α and HIF-2α 8. Currently, more than 2,500 HIF target genes have been identified based on the gene expression and chromatin immunoprecipitation (ChIP) techniques, such as ChIP-chip and ChIP-sequencing 9. These HIF target genes play the key roles in every critical aspect of “hallmarks of cancer”, including angiogenesis, cell survival, chemotherapy and radiation resistance, genetic instability, immortalization, immune evasion, metastasis, proliferation, metabolism, and stem cell maintenance 8. A growing body of evidence has improved the understanding of hypoxia-regulated cancer biology, including the communications mediated by small extracellular vesicles (sEVs) as one of the features.

In the past decade, there has been an increasing interest in the role of sEVs in cancer, mainly due to the discovery of functional molecular cargos in sEVs, which allow them to operate as signaling platforms for information delivery between cells 10. These functional molecules include proteins, nucleic acids, and metabolites. We and several other groups have provided direct evidence demonstrating an increased production of sEVs in response to hypoxia 11 and hypoxia-related conditions such as low pH 12 and oxidative stress 13. Hypoxia not only stimulates the production of sEVs, but also substantially alters the proteomic and nucleic acid profiles of sEVs 14. Tumor-derived sEVs, which are enriched in the tumor microenvironment, regulate tumoral signaling in both tumor and stromal cells. They play fundamental roles in various pathological scenarios, such as tumor invasiveness, angiogenesis, proliferation, chemotherapy and radiation resistance, immune evasion, metabolism, and cancer stemness 15.

Recent studies have shown that cancer cell-derived sEVs promote PMN formation via immunosuppression, angiogenesis, stromal cell remodeling, and oncogenic reprogramming 16, 17. However, the role and mechanism of sEVs in the regulation of hypoxia-mediated PMN formation remains poorly understood. A more recent study demonstrated that sEVs secreted by hypoxic prostate cancer cells promoted matrix metalloproteinase activity at PMNs 18. In the present study, we demonstrated that hypoxic HNSCC cell-derived sEVs induce cell invasion from the primary tumor and adhesion of non-hypoxic HNSCC cells on the second lesion site by delivering LOXL2. Hypoxic sEVs can prime PMN formation by stimulating FAK/Src signaling-dependent FN production by fibroblasts to recruit additional MDSCs in the PMN.

## Methods

Human and animal studies were approved by the Institutional Ethics Committee of Sichuan Cancer Hospital.

*Patients:* One hundred and thirty patients with newly diagnosed HNSCC (with no prior treatment) were enrolled in this study at Sichuan Cancer Hospital from May 2014 to September 2018. Exclusion criteria included recurrence at presentation; preoperative radiotherapy, chemotherapy, or hormone therapy; residual tumor at the surgical margin; and incomplete medical records. The formalin-fixed, paraffin-embedded samples from the patients were used for immunohistochemical analysis. All patients were informed about the investigative nature of the study and gave written informed consents before the study.

*Cell culture and hypoxia treatment:* Human HNSCC cell lines, SCC-9 and CAL-27, were obtained from and authenticated by ATCC. Cells were cultured in RPMI 1640 medium (Gibco, Grand Island, NY, USA) supplemented with 10% fetal bovine serum (HyClone, Logan, UT, USA), 2 mM L-glutamine, 25 mM 2-(4-(2-hydroxyethyl)-1-piperazinyl) ethanesulfonic acid (HEPES), 100 units/ml penicillin and 100 ug/ml streptomycin in a humidified 5% CO_2_ atmosphere. The cells were cultured under 20% O_2_ (normoxic) or 1% O_2_ (hypoxic) conditions, balanced with N_2_ in a 3-gas incubator (Binder, Tuttlington, Germany). MRC-5 cells were obtained from China Center for Type Culture Collection and were grown in minimum essential medium with Earle's balanced salts supplemented with 10% fetal bovine serum, 2 mM L-glutamine, 100 units of penicillin, and 100 μg/mL of streptomycin.

*Cloning and lentiviral packaging:* The Lenti-X shRNA expression system (Clontech, Mountain View, CA, USA) was used for the shRNA-mediated inhibition of HIF-1α and HIF-2α expression as described in our previous study 11. To knock down LOXL2 expression, shRNA against LOXL2 were annealed and cloned into the lentiviral vector pLenti-U6-pgkpuro. The targeting sequences were described previously 19. To overexpress LOXL2, human LOXL2 cDNA was amplified using primers Sense (5′-AAGCTTATGGAGAGGCCTCTGTG-3′) and antisense (5′-GATATCTTACTGCGGGGACAGCT-3′) and cloned into pLVX-puro vector as described previously 20. To KD vimentin, the validated siRNA sequences were transfected with 5 nM gene-specific Silencer^®^ Select siRNA (Thermo Fisher Scientific, Waltham, MA, USA), using Lipofectamine following the manufacturer's instructions.

The recombinant vectors were purified and co-transfected with Lenti-X HT packaging Mix (Clontech) into HEK 293T packaging cells. The virus-containing cell culture supernatants were collected 48 h after transfection, passed through a 0.45-μm filter, and stored at -80°C. Tumor cells were infected with recombinant lentivirus with 5 μg/ml polybrene, and stable clones were selected using 1.0 μg/ml puromycin 24 h after the infection.

*sEV isolation:* Before the isolation of sEV, cells were cultured in medium supplemented with 2% exosome-depleted fetal bovine serum (System Biosciences, CA, USA) for 48 h. The conditioned medium was centrifuged at 300 × g for 10 min, then at 2,000 × g for 10 min, and finally at 10,000 × g for 30 min to remove dead cells, cell debris and large particles. sEVs were isolated using the differential centrifugation/ultracentrifugation method as described previously 21. The conditioned medium and serum were harvested and centrifuged at 300 × g for 10 min at 4 °C to remove cellular debris. Then the supernatant was further centrifuged at 16,500 × g for 20 min at 4 °C and filtered through a 0.22 μm filter. Small EVs were then pelleted by ultracentrifugation at 120,000 × g (Optima™ MAX-XP Ultracentrifuge, Beckman Coulter) for 70 min at 4 °C. sEV pellets were resuspended in 0.2 μm-filtered PBS and stored at -80 °C until use. For in vivo tracking experiments, purified sEVs were fluorescently labeled with PKH26 membrane dye (Sigma). Labeled sEV were washed, collected by ultracentrifugation, and resuspended in PBS.

*DLS:* The size distribution of sEVs was determined as described previously 22. The capture settings and analysis settings were performed manually according to the manufacturer's instructions. Briefly, 10 μg protein of sEV preparation was shaken at 4 ℃ for 20 min and transferred to micro cuvettes for DLS measurement with a Zetasizer Nano ZS (Malvern Instruments Ltd., UK) at 20 ℃. DLS signal intensity was transformed to volume distribution [volume (%)]. Three technical replicates were carried out for each sEV sample.

*Electron microscopy:* EVs to be examined by SEM were isolated and loaded on to a carbon-coated electron microscopy grid. The samples were fixed with 2% glutaraldehyde and 2% paraformaldehyde in 0.1 M sodium cacodylate buffer at pH 7.3 for 3 h at room temperature. Samples were critical-point dried, mounted on specimen stubs, sputter-coated with 40 nm of gold/palladium, and visualized using a HITACHI S3400 scanning electron microscope (Tokyo, Japan).

*Quantitative real-time PCR (qRT-PCR):* Total RNA samples with a 260:280 nm absorbance ratio of ≥1.9 were reverse-transcribed using tan NCode™ VILO™ miRNA cDNA synthesis kit (Invitrogen, Carlsbad, CA, USA) following the manufacturer's instructions. Quantification of miRNA expression was performed using an NCode™ EXPRESS SYBR® GreenER™ miRNA qRT-PCR kit (Invitrogen) on an ABI PRISM 7300 sequence detection system (Applied Biosystems, Foster City, CA, USA). PCR conditions were 50 °C for 2min, 95 °C for 2min, and 40 cycles of amplification consisting of 95 °C for 15 s and 60 °C for 1min. Reactions were run in triplicate, and results were averaged.

*CCK-8 assay:* The proliferation of cells was determined using the CCK-8 kit, according to the manufacturer's instructions (Beyotime Biotechnology, Shanghai, China). Briefly, 2000 cells/well was seeded into a 96-well plate. 10 μL of CCK8 solution was added to each well and incubated in a humidified incubator (37 °C, 5% CO_2_) for 2 h. Absorbance (OD) at 450 nm was measured in a multi-well spectrophotometer.

*Invasion assay:* Cell invasion was assayed with a BioCoat Matrigel Invasion Chambers (Swedesboro, NJ, USA) according to the manufacturer's instructions. This invasion assay includes a cell culture insert with a 12-µm pore size polyethylene terephthalate membrane, coated with Matrigel matrix. Briefly, cells were seeded into the extracellular matrix layer, which had been previously rehydrated at room temperature for 1-2 h. Conditioned media (200 μl) [23]or sEVs (10 μg /ml) 21 were added to the lower chambers as a chemoattractant. Cells were incubated at 37 °C in a CO_2_ incubator (5% CO_2_) for 24 h. Invaded cells that had migrated to the bottom of the insert membrane were fixed in 4% paraformaldehyde and stained with 0.4% crystal violet. For quantification, crystal violet staining was dissolved in 1% SDS and optical density was measured at 550 nm using a microplate reader (Thermo Fisher Scientific, Waltham, MA, USA).

*Vybrant cell adhesion assay:*Role of sEV-treated fibroblasts on cancer cell adhesion was evaluated with a Vybrant cell adhesion assay kit (Thermo Fisher Scientific, Waltham, MA, USA) following the manufacturer's instructions. Briefly, sEV-treated MRC5 cells were seeded on microplates of the Vybrant cell adhesion assay and calcein acetoxymethyl ester (calcein AM)-stained Cal-27 cells were subsequently loaded at the concentration of 2 × 10^6^ cells/ml. After 1h-incubation, nonadherent calcein-labeled cells were washed out. Fluorescence from adherent cells was determined by a multi-well spectrophotometer.

*Chromatin immunoprecipitation (ChIP) assay:* ChIP assays were performed using a ChIP assay kit (Abcam) according to the manufacturer's instructions. Briefly, cells were fixed with formaldehyde, lysed, and sonicated to obtain DNA fragments in arranging in size from 200 to 1000 bp. Chromatin was then precipitated with non-specific IgG antibodies (Sigma, St. Louis, MO, USA), ChIP-grade mouse anti-HIF-1α(Abcam), or ChIP-grade rabbit anti-HIF-2α (Abcam). DNA was extracted and PCR was performed with primers specific for the LOXL2 promoter fragment containing a HIF response element (HRE) (fw, 5-CACACATACACGTGCACACA-3; rev, 5-AGGCTCTCCCCAAGGAAAT) 19 for 35 cycles 24. Primers that amplify a region in the promoter of miR-130b (hypoxia nonresponsive) were used as a negative control (fw: 5'-GCGAAACCCCAGCTCTACTA-3'; rv: 5'-ACACTCTCACTCTGTCGCCC-3') 25, while VEGF (fw: 5'-CAGGAACAAGGGCCTCTGTCT-3', rv: 5'-TGTCCCTCTGACAATGTGCCATC-3') 26 served as a positive control. The PCR products were resolved on a 1.5% agarose gel and visualized by ethidium bromide staining.

*Western blotting:* Total protein was isolated from sEVs and cells with a RIPA lysis and extraction buffer (Thermo Fisher Scientific, Waltham, MA, USA), and protein concentrations were detected by a BCA protein assay kit (Pierce, Rockford, IL, USA). Thirty micrograms of proteins from each sample were separated on an 8% SDS-PAGE gel and electrophoretically transferred to PVDF membranes (Millipore, Boston, MA, USA). Membranes were blocked with 2% BSA in TBS containing 0.1% Tween20 at 37 °C for 2 h and then incubated for 2 h with either mouse anti-HSP70 (1:1000, Santa Cruz, CA, USA), mouse anti-CD63 (1:1000, Santa Cruz), rabbit anti-albumin (1:1000, Santa Cruz), rabbit anti-E-cadherin (1:1000, Abcam), rabbit anti-N-cadherin (1:1000, Abcam), mouse anti-vimentin (1:1000, Abcam), rabbit anti-ZO-1 (1:1000, Abcam), rabbit anti-FAK (#3285, 1:1000, Cell Signaling Technology, Danvers, MA, USA), anti-p-FAK (Y397, #3283, 1:1000, Cell Signaling Technology), rabbit anti-Src (#2108, 1:1000, Cell Signaling Technology), rabbit anti-p-Src (Tyr416, #2101, 1:1000, Cell Signaling Technology), rabbit anti-fibronectin (1:1000, Abcam), or rabbit anti-β-actin (1:1000, NOVUS, Littleton, CO, USA) antibodies. Horseradish peroxidase-conjugated anti-mouse or anti-rabbit IgG was used as a secondary antibody (diluted 1:5000 in TBST with 2% BSA and incubated for 1 h). Bands were scanned using a densitometer (GS-700; Bio-Rad Laboratories, Hercules, CA, USA), and quantification was performed using the Quantity One 4.4.0 software.

*iTRAQ:* Total proteins were extracted from sEVs and quantified. iTRAQ labeling and LC MS/MS analyses were performed by PTM BIO (Hangzhou, Zhejiang, China). Briefly, the proteins were digested with trypsin at 37°C overnight. Then the peptides were reconstituted in 0.5 M TEAB and labeled using an iTRAQ 8-plex kit (Applied Biosystems, CA, USA) according to the manufacturer's manual. Each group of the peptide segments was labeled with different iTRAQ tags at room temperature for 3h. Then samples were pooled and processed by separation and mass spectrometric analysis. Samples were analyzed using an Ulti-Mate 3000 RSLC nano LC system (Thermo Fisher Scientific) coupled to an LTQ-Orbitrap mass spectrometer (LTQ-Orbitrap, Thermo Fisher Scientific).

*Flow cytometry:* Single-cell suspensions were prepared from the lungs. Red cells were removed using ammonium chloride lysis buffer when necessary. 1 × 10^6^ cells were incubated at 4 ℃ for 30 min with FITC-anti-Gr-1 antibody for 30 min. GFP+ cancer cells and Gr-1-stained cells were analyzed using a BD FACS Canto II flow cytometer. Data were analyzed by FlowJo V10.

*Enzyme-linked immunosorbent assay (ELISA):* LOXL2 protein contents in serum sEVs were measured by enzyme-linked immunosorbent assay (ELISA) using a Human LOXL2 ELISA Kit (Abcam, Cambridge, MA, USA), following the manufacturer's instructions. The intensity of color was measured at 450 nm in a microtest plate spectrophotometer. Each sample was tested in triplicate wells and was normalized to standard curves generated for each set of samples assayed.

*Xenograft:* The Nude mice (females, 6 weeks of age) were obtained from Charles River (Beijing, China). PKH67 -labeled sEVs (5 mg) were i.v. injected into the tail vein of mice twice a week for 6 weeks. Fibronectin (Sigma) was converted to soluble fibronectin (sFN) by mixing 100 μg of fibronectin in 100 μl PBS. The mice were treated with i.p. injections of sFN (200 μg) in PBS twice a week for 6 weeks as described previously 27. Mice were treated daily by oral gavage with 70 mg/kg BAPN (Sigma) in 5% DMSO/2.5% Tween20 in water as described previously 28. Then tumor cells were i.v. injected (5×10^6^ cells/200 μl PBS/mouse) into the tail vein of the mice. One week later, mice were euthanized and lung tissues samples were analyzed by flow cytometry and frozen sliced for florescence microscopic observation. For xenograft growth, tumor cells were s.c. injected (5×10^6^ cells/200 μl PBS/mouse) into the back of nude mice after 6 weeks of sEV injection. The tumor size was monitored weekly by measuring the diameters using Vernier calipers, and calculated as πls^2^/6, where l = the long side and s = the short side. Mice were euthanized at week 7.

*Immunofluorescence (IF) and immunohistochemistry (IHC):* The formalin-fixed paraffin-embedded sections were deparaffinized and rehydrated, the endogenous peroxidase was blocked, and the antigen was retrieved. Then the slides were incubated at 37 °C with mouse anti-HIF-1α (1:200, NOVUS, Littleton, CO, USA), mouse anti-HIF-2α (1:100, Abcam, Cambridge, MA, USA), and rabbit anti-LOXL2 (1:100, Abcam) for 2 h. The slices were then incubated with biotinylated goat anti-mouse or goat anti-rabbit IgG for 1 h, and with streptavidin-peroxidase for 30 min. The 0.02% diaminobenzidine tetrahydrochloride (DAB, 0.02%) was used as a chromogen, and the slides were counterstained with haematoxylin. The percentage of the positive cells was estimated using an image analysis system (Leica, Germany).

Frozen lung tissue samples were embedded in optimal cutting temperature compound (OCT, Sakura Finetek, Tokyo, Japan), frozen at -80℃, and cut (6 µm) in a cryostat (Leica Microsystem, Bannockburn, IL, USA). Frozen sections were dried at 20 ℃ for 1 h, fixed in acetone at 4 ℃ for 20 min, air-dried for 10 min at 20 ℃, and incubated in 20% goat serum at 37 ℃ for 30 min to block nonspecific binding. For IF double staining of vimentin and E-cadherin, slides were incubated with rabbit anti-E-Cadherin (1:100, Abcam) and mouse anti-vimentin (1:100, Abcam), and rabbit monoclonal anti-Cytokeratin 10 (1:100, Abcam) for 2 hours. Then slides were incubated for 1 hour with Alexa Fluor 488 goat anti-mouse IgG (1:500, Invitrogen) and Alexa Fluor 594 goat anti-rabbit IgG (1:500, Invitrogen). For S100A4, CD31, and CK IF staining, slides were washed, and incubated for 1 h with Alexa Fluor 594 goat anti-rabbit IgG (1:500, Invitrogen). After washing, the coverslips were counter-stained with 4', 6-diamidino-2-phenylindole (DAPI; 1 μg/μL), and examined using a Leica DMI6000 B fluorescence microscope (Leica Microsystem) with Leica FW4000 V1.0 software.

*Statistics:* The comparisons of the mean values between the groups were performed by one-way ANOVA, and the Dunn's multiple comparison test was further used to determine significant differences between the groups. The comparison of the mean values between 2 groups was performed using student's T test. The power calculation was performed with an online calculation tool (DSS Research, https://www.dssresearch.com/resources/calculators). In the comparison of serum sEV LOXL2 between groups, an overall sample of 130 achieved at least 86.7% power. The survival curves were estimated using the Kaplan-Meier method and differences between groups were compared using the log-rank test. A Cox proportional hazards model was applied to identify prognostic variables that predict overall survival. A value of *P* < 0.05 was considered statistically significant. All statistical analyses were performed using the SPSS package (version 18.0, IBM, Armonk, New York, USA).

## Results

### sEVs mediate hypoxic cancer cell-induced cell invasion

**Figure 1:**
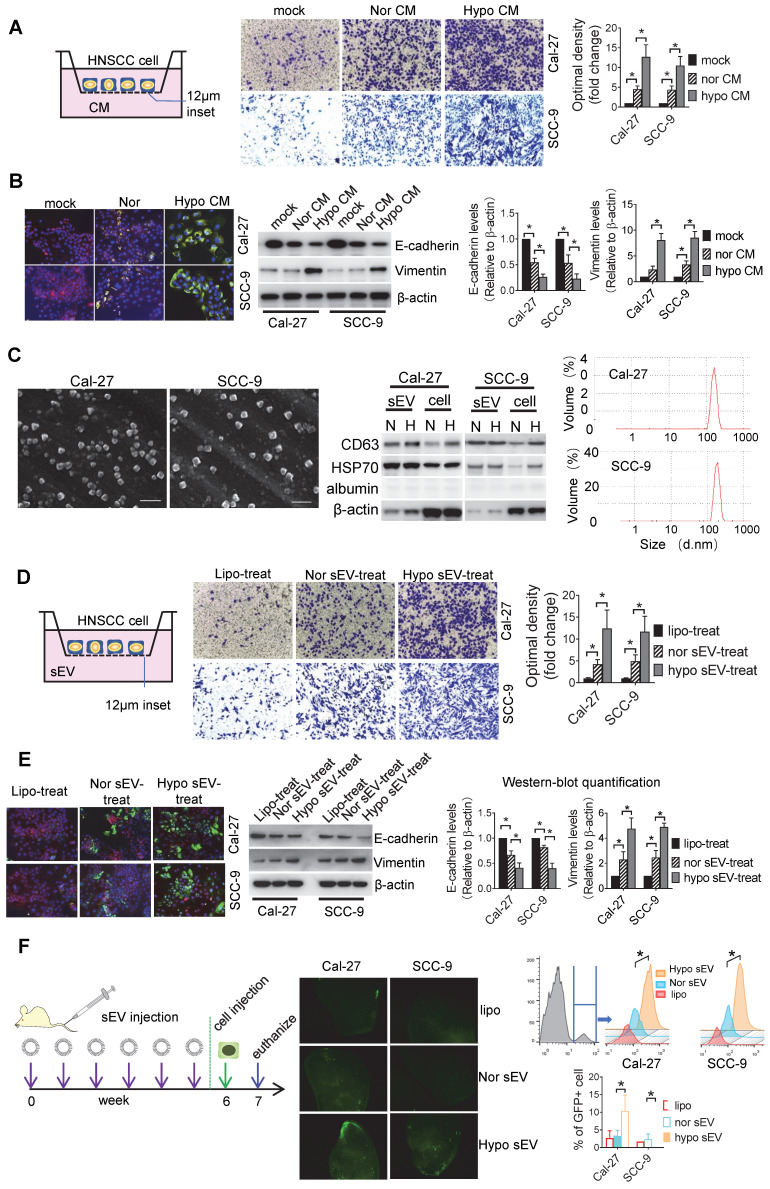
** sEVs mediate hypoxic cancer cell-induced normoxic cell invasion.** (A) Invasion assays were performed with Cal-27 and SCC-9 cells seeded on 12μm insets which were placed on 24-well plates filled with CM from normoxic (Nor) and hypoxic (Hypo) Cal-27 and SCC-9 cells respectively. PBS added on the wells served as mock control (upper left panel). Cells that migrated to the bottom surface were stained with crystal violet and observed by light microscopy (Lower panel, magnification, 200). Quantitative analysis of crystal violet optical density (Upper right panel). Data represent at least three experiments performed in triplicate. * *P* < 0.05; (B) Normoxic and hypoxic CM-treated Cal-27 and SCC-9 cells were measured for E-cadherin and vimentin expression by immunofluorescence staining (upper left panel) and Western blotting (upper right panel). Quantification of Western blotting (lower panel). Data represent at least three experiments performed in triplicate. * P < 0.05; (C) sEVs were measured by SEM (left), Western-blotting (middle), and DLS (right panel) N: normoxia, H: hypoxia. (D) An invasion assay was performed with Cal-27 and SCC-9 cells seeded on 12μm insets which were placed on 24-well plates filled with sEVs derived from Cal-27 and SCC-9 cells respectively. Liposome (Lipo) was used as a negative control. Cells that migrated to the bottom surface were stained with crystal violet and observed by light microscopy (Lower panel, magnification, 200). Quantitative analysis of crystal violet optical density (Upper right panel). Data represent at least three experiments performed in triplicate. * *P* < 0.05; (E) Normoxic and hypoxic sEV-treated Cal-27 and SCC-9 cells were measured for E-cadherin and vimentin expression by immunofluorescence staining (upper left panel) and Western blotting (upper right panel). Quantification of Western blotting were performed relative to β-actin (lower panel). Data represent at least three experiments performed in triplicate. * *P* < 0.05; (F) Hypoxic or normoxic sEVs were i.v. injected to nude mice twice a week. After 6-week injection, GFP+ tumor cells were i.v. injected. One week later, mice were euthanized and lung tissues were frozen sliced for florescence microscopic observation (middle panel). The GFP+ cells were quantified by flow cytometry (right panel), n=6, * *P <* 0.05

Hypoxia is a known driving force of cell invasiveness. Since cells communicate with each other, we investigated whether hypoxic cells can induce invasiveness in normoxic cells. We performed an invasion assay with HNSCC cells seeded into the Matrigel-coated inserts and conditioned media (CM) of normoxic and hypoxic HNSCC added to the lower chamber. Hypoxic CM significantly stimulated the invasion of Cal-27 (*P* = 0.002) and SCC-9 (*P* = 0.01) cells compared with the effect of normoxic CM **(Figure** 1A). Hypoxic CM induced a significant increase in vimentin and a decrease in E-cadherin expression in two cell lines according to the data of immunofluorescence staining and Western blotting (**Figure** 1B). This was validated by ZO-1 and N-cadherin Western blotting, suggesting that hypoxic HNSCC CM can elicit invasiveness in normoxic cells (**Figure** S1A). Since CM contains large number of sEVs secreted by the cancer cells, we investigated whether hypoxic CM-induced cell invasion was dependent on sEVs. Cal-27 and SCC-9 cell-derived sEVs that showed bona fide characteristics of sEVs were isolated. The sEVs displayed a cup-shaped appearance under scanning electron microscopy (SEM), enriched in CD63 and HSP70 but not in albumin. The sizes of the sEVs ranged from 50 to 200 nm, as visualized by Dynamic light scattering (DLS) (**Figure** 1C). We observed that the release of sEVs by both Cal-27 and SCC-9 cell lines was induced by hypoxia, as determined by sEV protein concentration (**Figure S1**B). Equal amounts of hypoxic and normoxic sEVs were added to the lower chamber of the invasion assay with Cal-27 and SCC-9 cells seeded on the inserts. Hypoxic Cal-27 (*P* = 0.017) and SCC-9 (*P* = 0.04) cell-derived sEVs significantly induced the invasiveness of the Cal-27 and SCC-9 cells, respectively (**Figure** 1D). Significant increases in vimentin (**Figure** 1E) and N-cadherin (**Figure** S1C) expression and decreases in E-cadherin (**Figure** 1E) and ZO-1 (**Figure** S1C) expression was observed in hypoxic sEV-treated Cal-27 and SCC-9 cells compared with that in normoxic sEV-treated cells. Cells cultured under hypoxia had significantly increased vimentin expression but had equal levels of vimentin in their sEVs (**Figure S**1D). Additionally, vimentin knockdown (KD) in HNSCC cells did not affect the release of sEVs (**Figure S**1E). Moreover, cells treated with sEVs derived from vimentin KD cells had equal invasiveness (**Figure S**1F) and levels of epithelial-to-mesenchymal transition (EMT) markers (**Figure S**1G) to those treated with sEVs derived from wild-type cells. These results suggest that the hypoxic sEV-induced invasion and vimentin expression in recipient cells was not dependent on vimentin carried by sEVs.

After invasion into the circulation, cancer cells need to colonize at distant organs to form a metastatic lesion. However, before the formation of metastatic lesions, the primary tumors can educate the secondary sites to form a supportive metastatic environment, termed PMN 29. To test whether hypoxic tumor cell-derived sEVs can contribute to PMN formation, we intravenously (i.v.) injected hypoxic or normoxic sEVs into nude mice twice a week. After 6 week of injection, GFP+ tumor cells were i.v. injected. One week later, the mice were euthanized and the lung tissue samples were analyzed by flow cytometry. Furthermore, fluorescence microscopy was performed on the frozen sections. Hypoxic sEV-treated mice showed a significant increase in colonized tumor cells in the lung compared with that in normoxic sEV-treated mice (*P* < 0.001, **Figure** 1F). Three weeks after the cell injection, metastatic lesions were formed as determined by IF staining of cytokeratin 10 which was strongly expressed in the HNSCC cells rather than in normal lung tissues (**Figure** S1H). These results suggest that hypoxic tumor cell-derived sEVs may drive normoxic cells to EMT and contribute to the formation of PMN.

### Protein expression profile of sEVs

Proteins represent one of the most important cargos within sEVs which are involved in hypoxia regulation. We sought to investigate the protein expression profile of sEVs derived from normoxic and hypoxic HNSCC cells using Isobaric Tagging Technology for Relative Quantitation (iTRAQ). The HNSCC cell line Cal-27 was cultured under 20% O_2_ and 1% O_2_. After 24 h of treatment, sEVs were isolated from the supernatant, and total protein was prepared for iTRAQ analysis.

**Figure 2:**
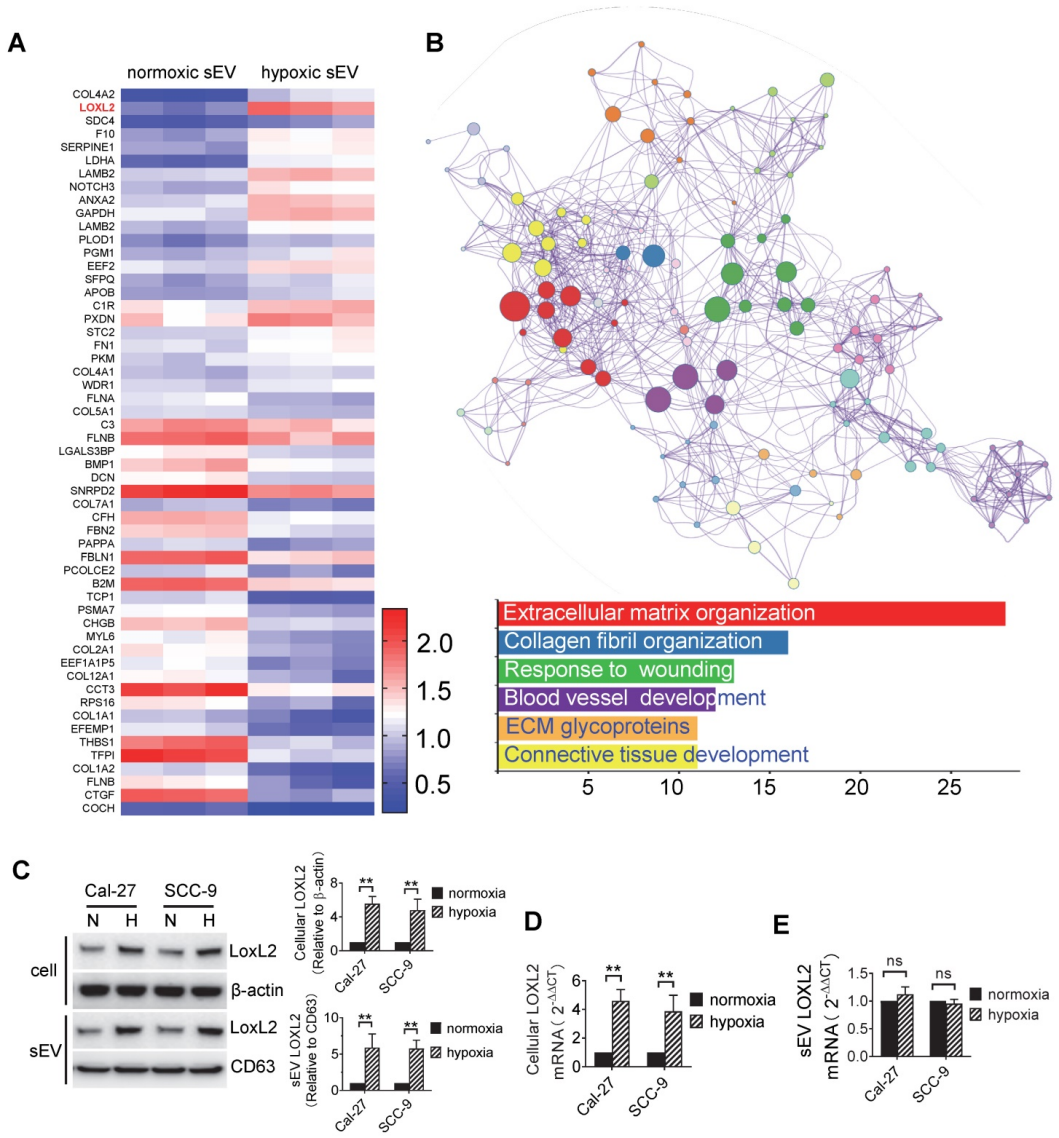
** Protein expression profiles of sEVs.** (A) Heatmap diagram of differential protein expression between normoxic and hypoxic sEVs derived from Cal-27 cells. Protein expression data were obtained using iTRAQ. Expression values shown are mean centered. Red, increased expression, blue, decreased expression, and white, mean value; (B) Functional and pathway enrichment analyses of the differently expressed proteins were performed using Metascape with express analysis. The network is visualized using Cytoscape, where each node represents an enriched term and is colored first by its cluster ID. Top 5 enriched clusters were list with bar graph; (C) LOXL2 protein expression levels in normoxic and hypoxic Cal-27 and SCC-9 cells and their sEVs were analyzed by Western blotting. Cellular LOXL2 protein expression was quantified relative to β-actin and sEV LOXL2 protein levels were quantified relative to CD63. Data represent at least three experiments performed in triplicate. H: hypoxia, N: normoxia. ** *P* < 0.001; (D) LOXL2 mRNA levels in cells were measured by qRT-PCR. Data represent at least three experiments performed in triplicate. ** *P* < 0.001; (E) LOXL2 mRNA levels in sEVs were measured by qRTPCR. Data represent at least three experiments performed in triplicate. ns: non-significant.

Initially, a total of 576 proteins were identified in normoxic and hypoxic sEVs. Identified protein pathways were annotated by Kyoto Encyclopedia of Genes and Genomes (KEGG), which showed that extracellular matrix (ECM)-receptor interaction was among the most important pathways (**Figure S2**A). The identified proteins were further classified by Gene Ontology (GO) annotation into three categories: biological process, cellular compartment, and molecular function (**Figure S**2B). Binding turned to be the most important function (52%) of the identified proteins (**Figure S**2B, bottom panel). Using *P* < 0.05 as the cutoff threshold, 55 proteins were defined as significantly differentially expressed between normoxic and hypoxic sEVs (**Figure 2**A, **Table** s1). These differentially expressed proteins (DEPs) include 23 proteins that were upregulated in hypoxic sEVs versus normoxic sEVs. Functional and pathway enrichment analyses of the DEPs were performed using Metascape 30. DEPs between normoxic and hypoxic sEVs were mainly enriched in extracellular matrix organization, collagen fibril organization, wounding responses, blood vessel development, ECM glycoproteins, and connective tissue development (**Figure** 2B).

In the DEPs, we focused on LOXL2 for subsequent studies because LOXL2 expression was significantly increased in hypoxic sEVs with the highest fold increase and is involved in the regulation of the top function and pathways including extracellular matrix organization 31, collagen fibril organization 32, wounding responses 33, and blood vessel development 34. LOXL2 expression in Cal-27 and SCC-9 cells and their sEVs were validated by Western blotting. We showed that LOXL2 protein levels were significantly increased in hypoxic cells and sEVs compared with that in the normoxic controls (**Figure** 2C). qRT-PCR validated a significant increase in LOXL2 mRNA in hypoxic Cal-27 and SCC-9 cells (**Figure** 2D) but not in their sEVs (**Figure** 2E). These results suggest that the potential role of LOXL2 shuttled by sEV should be dependent on LOXL2 protein rather than mRNA.

### LOXL2 mediated hypoxic sEV-induced cell invasiveness

**Figure 3:**
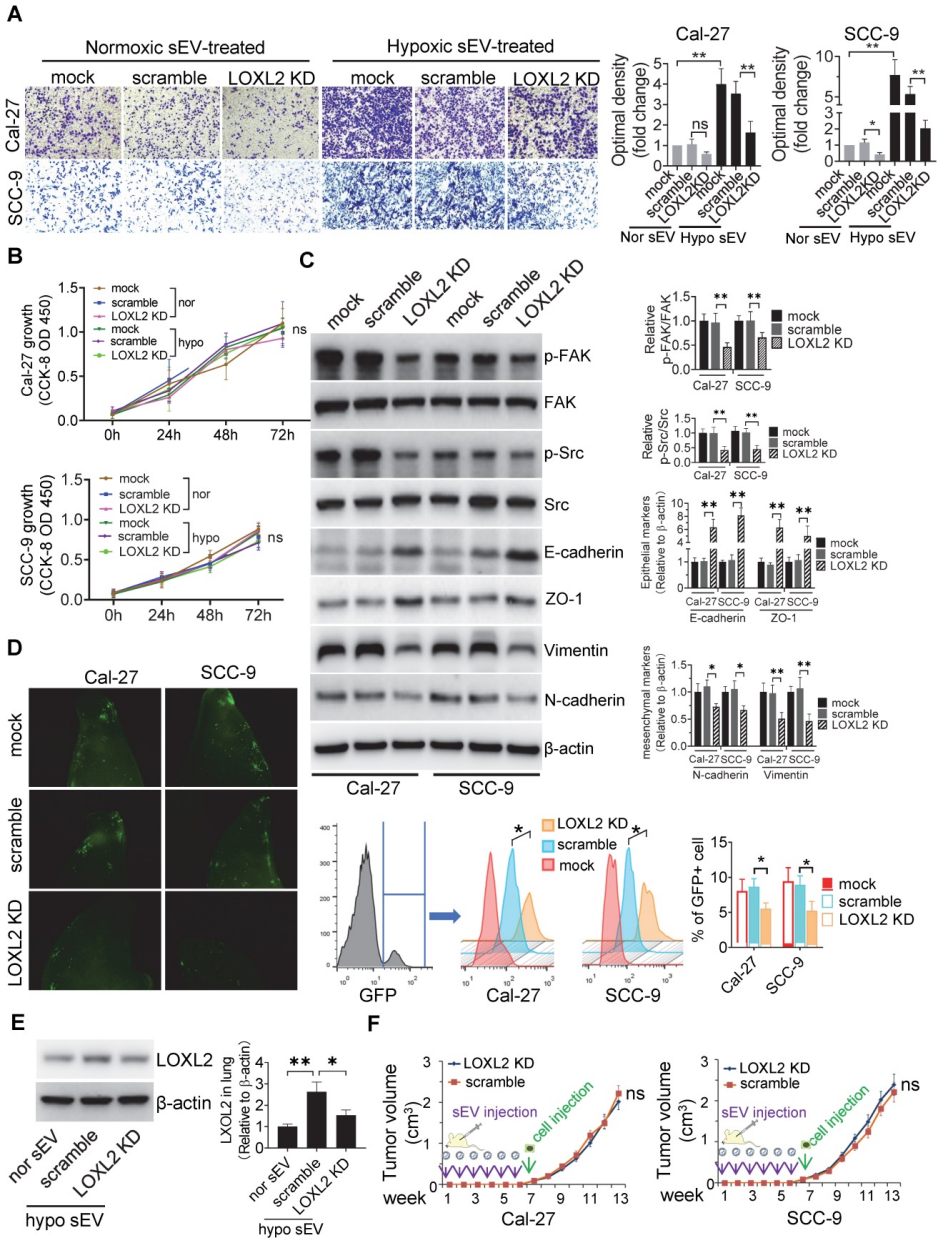
** LOXL2 mediated hypoxic sEV-induced cell invasiveness.** (A) Invasion assays were performed with Cal-27 and SCC-9 cells seed on 12μm insets which were placed on 24-well plates filled with sEVs derived from wild-type and LOXL2 KD Cal-27 and SCC-9 cells respectively. Cells that migrated to the bottom surface were stained with crystal violet and observed by light microscopy (Lower panel, magnification, 200). Quantitative analysis of crystal violet optical density (Upper right panel). Data represent at least three experiments performed in triplicate. * *P* < 0.05, ** *P* < 0.001, ns: non-significant; (B) Cal-27 (upper panel) and SCC-9 (lower panel) cells were treated with sEVs derived from wild-type and LOXL2 KD cells respectively. The proliferation of sEV-treated OSCC cells was measured by a CCK-8 assay. Experiments were performed in triplicate. ns: non-significant; (C) Western blotting was performed to evaluate the expression of p-FAK, FAK, p-Src, Src, Ecadherin, ZO-1, vimentin, and N-cadherin expression levels in Cal-27 and SCC-9 cells treated with wild-type and LOXL2 KD sEVs. Quantification of Western blotting was performed relative to β-actin. Data represent at least three experiments performed in triplicate. ** *P* < 0.001; (D) Wild-type and LOXL2 KD sEVs were i.v. injected to nude mice twice a week for 6 weeks. Then GFP+ tumor cells were i.v. injected. One week later, mice were euthanized and lung tissues were frozen sliced for florescence microscopic observation (middle panel). The GFP+ cells were quantified by flow cytometry (right panel), n=6, * *P <* 0.05; (E) The expression of LOXL2 in mice lungs were quantified by Western blotting. n=6, * P < 0.05, ** P < 0.001 (F) Cal-27 (left) and SCC-9 (right) cells were s.c. injected into the back of nude mice after 6 weeks of in situ sEV injections. Growth of xenograft tumors were monitored weekly. ns: non-significant

To study the function of sEV-LOXL2, we knocked down LOXL2 in Cal-27 and SCC-9 cells with lentiviral shRNA which achieved >70% downregulation of LOXL2 mRNA in cells as measured by qRT-PCR (**Figure S3**A). LOXL2 KD resulted in a significant decrease in LOXL2 protein levels in sEVs as well as in their parent cells (**Figure S**3B). LOXL2 KD in Cal-27 cells did not affect the release of sEVs (**Figure S**3C). The growth of cells was not affected by LOXL2 KD (**Figure S**3D), while the invasion of cells was significantly inhibited by LOXL2 KD under both normoxia and hypoxic conditions (**Figure S**3E). To investigate the effect of LOXL2 KD on sEV-treated recipient cells, we treated cells with sEVs derived from wild-type and LOXL2 KD cells which were cultured under hypoxic conditions. sEVs derived from wild-type hypoxic HNSCC cells significantly increased LOXL2 protein rather than mRNA levels in the recipient cells (**Figure S**3F). Hypoxic sEV-induced Cal-27 (*P* = 0.002) and SCC-9 (*P* = 0.006) cell invasion was significantly abrogated by LOXL2 KD **(Figure 3**A). The proliferation of sEV-treated HNSCC cells was measured by the CCK-8 assay, which showed no significant difference in proliferation between the scrambled and LOXL2 KD groups (**Figure** 3B). These results suggest that hypoxia-induced LOXL2 contributes to the invasion rather than to the proliferation of HNSCC cells; sEVs derived from hypoxic cancer cells can deliver increased LOXL2 protein to non-hypoxic cells to achieve a pseudo-hypoxia behavior.

LOXL2 KD significantly increased the expression of E-cadherin and decreased the expression of vimentin in hypoxic cells rather than in normoxic cells (**Figure S**3G). We then investigated whether LOXL2 delivered by sEVs could regulate EMT in recipient HNSCC cells. Western blotting showed that cells treated with sEVs derived from hypoxic LOXL2 KD HNSCC cells had remarkably decreased expression of vimentin and N-cadherin and increased E-cadherin and ZO-1 levels compared with those treated with wild-type sEVs (**Figure** 3C). Meanwhile, LOXL2 KD sEV-treated cells had decreased phosphorylation of both FAK and Src, which are the components of the key signaling pathway regulating EMT (**Figure** 3C). To investigate the direct regulation of LOXL2 on FAK and Src phosphorylation, we measured the expression of p-FAK and p-Src in normoxic and hypoxic cells with or without LOXL2 KD. In accordance with sEV-treated cells, cells cultured under hypoxia had significantly increased phosphorylation of FAK and Src, which was significantly abrogated by LOXL2 KD (**Figure S4**A). These results suggest that hypoxic sEVs delivering LOXL2 could induce the EMT and activation of FAK/Src signaling in non-hypoxic recipient HNSCC cells.

We then investigated the role of FAK/Src signaling in mediating LOXL2-induced EMT and invasion of HNSCC cells. Cal-27 and SCC-9 cells were cultured under hypoxic condition in the presence of a FAK (PF-562271) or Src inhibitor (Src inhibitor-1). PF-562271 and Src inhibitor-1 significantly inhibited both p-FAK and p-Src levels in hypoxic Cal-27 and SCC-9 cells (**Figure S**4B). We then overexpressed LOXL2 in normoxic Cal-27 and SCC-9 cells, which resulted in a remarkable increase in cell invasion (**Figure S**4C). The LOXL2 overexpression (OE)-induced cell invasion was significantly inhibited by both PF-562271 and Src inhibitor-1 (**Figure S**4C). Consistently LOXL2 OE induced vimentin expression and reduced E-cadherin expression, which was significantly reversed by FAK and Src inhibitors (**Figure S**4D). These results suggest that LOXL2 could induce the EMT and invasion of HNSCC cells by activating FAK/Src signaling.

We then investigated whether LOXL2 mediates hypoxic sEV-induced tumor cell colonization in the lung. Fluorescence microscopy and flow cytometry (**Figure** 3D) showed that LOXL2 KD sEV-treated mice had significantly reduced numbers of GFP+ HNSCC cells in the lung compared with those in the scramble control (Cal-27, *P* = 0.003; SCC-9, *P* = 0.01). This was accompanied by decreased LOXL2 levels in lung tissues of mice treated with LOXL2 KD sEVs (**Figure** 3E).

To evaluate the contribution of sEV-LOXL2 on primary tumor growth, we subcutaneously injected Cal-27 and SCC-9 cells into the backs of nude mice after 6 weeks of sEV injection. LOXL2 KD had no significant effect on the xenograft tumor growth of Cal-27 or SCC-9 cells (**Figure** 3F) cells. These in vivo data supported the ex vivo results, confirming that hypoxic sEVs that carry an increased amount of LOXL2 could contribute to target cell invasion and to the formation of a PMN in the second lesion.

### LOXL2 mediated cell invasion is HIF-1α and HIF-2α-dependent

**Figure 4:**
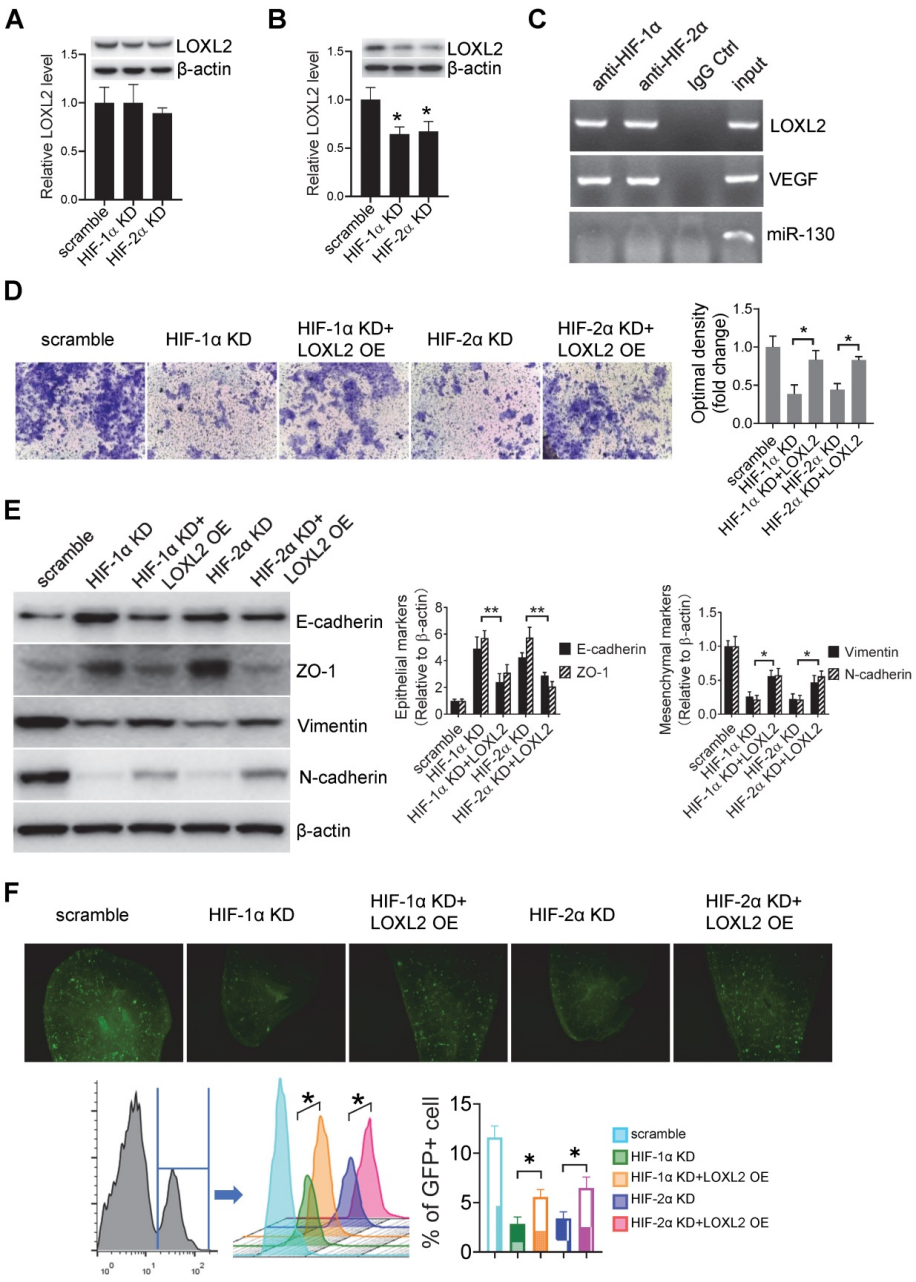
** LOXL2 mediated cell invasion is HIF-1α and HIF-2α dependent.** (A, B) Western-blotting and qRT-PCR were performed respectively to evaluate the protein and mRNA levels of LOXL2 in wild-type and HIF-1α/HIF-2α KD cells that were cultured under normoxic (A) and hypoxic (B) conditions. Data represent at least three experiments performed in triplicate * P < 0.05. (C) ChIP assays were performed to evaluate the binding of HIF-1α and HIF-2α on the promoter of LOXL2 gene. VEGF: positive control. miR-130: negative control. (D) Invasion assays were performed on Cal-27 cells which were treated by sEVs derived from hypoxic HIF-1α and HIF-2α KD Cal-27 cells. Left panel, cells that migrated to the bottom surface were stained with crystal violet and observed by light microscopy (magnification, 200). Right panel, quantitative analysis of crystal violet optical density. Data represent at least three experiments performed in triplicate. * *P <* 0.05; (E) Expression of mesenchymal markers (vimentin and N-cadherin) and epithelial markers (E-cadherin and ZO-1) in sEV-treated Cal-27 cells were measured by Western blotting. Quantification of Western blotting was performed (right panel). Data represent at least three experiments performed in triplicate. * *P <* 0.05, ** *P <* 0.001 (F) sEVs derived from wild-type and HIF-1α/HIF-2α KD cells were i.v. injected to nude mice twice a week for 6 weeks. Then GFP+ tumor cells were i.v. injected. Mice were euthanized and lung tissues were frozen sliced for florescence microscopic observation (upper panel) and quantified by flow cytometry (lower panel). n=6, * P < 0.05

To determine whether hypoxia-induced LOXL2 expression is HIF-1α- or HIF-2α-dependent, a stable KD of HIF-1α and HIF-2α was performed in Cal-27 cells using lentiviral shRNAs. Under the normoxic conditions, LOXL2 mRNA and protein levels in the cells were not significantly influenced by HIF-1α or HIF-2α KD (**Figure 4**A). However, under the hypoxic conditions, LOXL2 mRNA and protein levels were significantly decreased by HIF-1α (*P* = 0.012) and HIF-2α (*P* = 0.018) KD (**Figure** 4B). To validate the binding of HIF-1α and HIF-2α to the predicted HRE region of LOXL2, we performed a ChIP assay. PCR products corresponding to the HRE-containing region of LOXL2 were detected in cells cultured under hypoxic conditions after HIF-1α and HIF-2α immunoprecipitation (**Figure** 4C). VEGF, which is the validated target gene of both HIF-1α and HIF-2α, served as a positive control. The miR-130 promoter region, which does not contain an HRE, did not show any detectable recruitment of HIF-1α or HIF-2α under hypoxia (**Figure** 4C). These results confirmed the direct binding of HIF-1α and HIF-2α to the HRE region of LOXL2 upon exposure to hypoxia.

Invasion assay showed that cells treated with sEVs derived from hypoxic HIF-1α (*P* < 0.001) and HIF-2α KD (*P* < 0.001) cells had decreased invasiveness compared with that of the cells treated with sEVs derived from the scramble control cells (**Figure** 4D). This decrease was restored by overexpression of LOXL2 in HIF-1α (*P* = 0.003) and HIF-2α KD (*P* = 0.009) sEVs (**Figure** 4D), suggesting that LOXL2 mediates the HIF-1α/HIF-2α-induced invasiveness of HNSCC cells. WB showed that HIF-1α and HIF-2α KD sEVs inhibited mesenchymal markers (vimentin and N-cadherin) expression and increased epithelial markers (E-cadherin and ZO-1) expression, and this effect was reversed by LOXL2 OE (**Figure** 4E).

Pre-treatment of mice with sEVs derived from hypoxic HIF-1α (*P* = 0.03) and HIF-2α (*P* = 0.01) KD cells decreased the colonization of GFP+ cells in the lung compared with that detected in the case of sEVs derived from the scramble control cells (**Figure** 4F). Colonization was restored by LOXL2 OE in the HIF-1α and HIF-2α KD sEVs as determined by fluorescence microscopy and flow cytometry (**Figure** 4F).

These results suggest that hypoxic sEVs induced HNSCC cell invasion and pre-metastatic colonization were regulated by LOXL2 in a HIF-1α- and HIF-2α-dependent manner.

### sEV-LOXL2 induced pre-metastatic niche formation via FN

**Figure 5:**
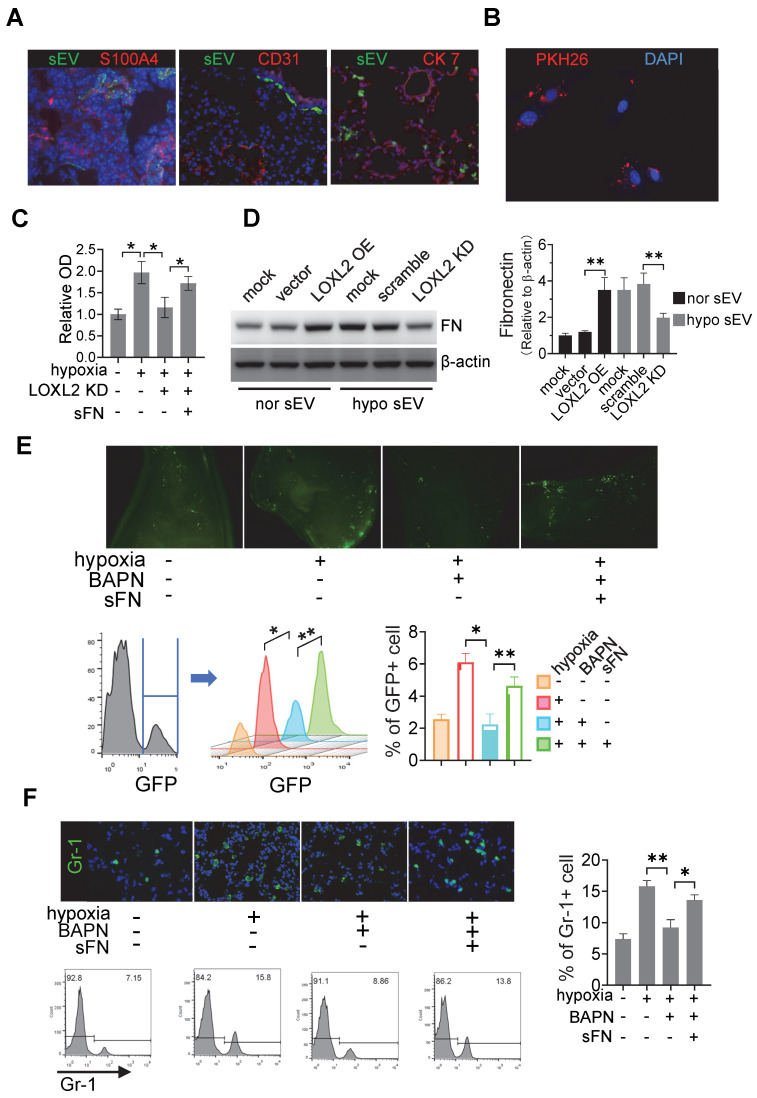
** sEV-LOXL2 induced pre-metastatic niche formation via FN.** (A) PKH67 labelled sEVs from Cal-27 cells were i.v. injected into nude mice. Mice were euthanized and lung tissues were frozen sliced for immunofluorescence staining of S100A4, CD31, and CK7; (B) MRC-5 cells were incubated with PKH26-labeled sEVs and observed under fluorescence microscopy for the internalization; (C) MRC-5 cells were treated with normoxic sEVs, hypoxic sEV, BAPN, and soluble fibronectin. sEV-treated MRC5 cells were seeded on microplates of Vybrant cell adhesion assay with calcein AM-stained Cal-27 cells loaded subsequently. Experiments were performed in triplicate. * *P* < 0.05; (D) Western blotting was performed to evaluate the expression of FN in MRC-5 cells treated by sEVs derived from wild-type and LOXL2 KD Cal-27 cells which were cultured under either normoxic or hypoxic conditions. Quantification of Western blotting was performed (right panel). Data represent at least three experiments performed in triplicate. ** *P <* 0.001; (E) mice received i.v. injection of normoxic sEVs, hypoxic sEV, LOXL2 inhibitor BAPN, and soluble fibronectin. GFP+ Cal-27 cells were then i.v. injected. The colonization of GFP+ cells in the lung were quantified by flow cytometry (lower panel) and observed by florescence microscopy (upper panel). n=6, * *P <* 0.05, ** *P <* 0.001; (F) Gr-1+ myeloid cell infiltration in the sEV-treated lungs were measured by IF staining of Gr-1(upper left). The Gr-1 infiltration was quantified by flow cytometry (lower left and right panels). n=6, * *P* < 0.05, ** *P <* 0.001.

To identify the cells that uptake tumor sEVs in the pre-metastatic organ, we intravenously injected PKH67-labeled sEVs from Cal-27 cells into mice. PKH67+ sEVs are mainly co-localized with S100A4-positive fibroblasts rather than with CD31-positive endothelial cells or cytokeratin 7 (CK7) positive lung epithelial cells (**Figure 5**A). To evaluate whether sEVs can be internalized by fibroblasts, we cultured MRC5 cells and treated them with PKH26-stained sEVs derived from Cal-27 cells. PKH26-labeled sEVs could be internalized by MRC5 cells during the incubation period as determined by fluorescence microscopy (**Figure** 5B, supplementary video).

To investigate whether LOXL2-enriched sEV-treated fibroblasts affect tumor cell adhesion, we treated MRC5 cells with sEVs derived from normoxic Cal-27 cells, sEVs derived from hypoxic Cal-27 cells with or without LOXL2 KD. Soluble fibronectin (sFN) was added to restore extracellular FN. sEV-treated MRC5 cells were seeded on microplates of the Vybrant cell adhesion assay and calcein acetoxymethyl ester (calcein AM)-stained Cal-27 cells were subsequently loaded. The adhesion assay demonstrated that hypoxic HNSCC sEV-treated fibroblasts induced the adhesion of HNSCC cells (*P* = 0.01), which was inhibited by LOXL2 KD (*P* = 0.005, **Figure** 5C). The LOXL2 KD-inhibited cell adhesion was restored by the administration of sFN (*P* = 0.038, **Figure** 5C). sEVs derived from normoxic LOXL2 OE Cal-27 cells induced the production of FN by MRC5 cells. Hypoxic sEVs carrying downregulated LOXL2 failed to induce FN production compared with wild-type hypoxic sEVs (**Figure** 5D). In vivo colonization experiments confirmed the results of the in vitro adhesion assay, showing that hypoxic sEV-induced colonization of HNSCC cells was inhibited by BAPN, a LOXL2 inhibitor (*P* = 0.02). This inhibition was rescued by sFN according to the results of fluorescence microscopy and flow cytometry (*P* = 0.03, **Figure** 5E).

LOXL2 OE Cal-27 cells were cultured under normoxic conditions, while LOXL2 KD cells were cultured under hypoxic conditions. Equal amounts of sEVs obtained from these cells were added to MRC5 fibroblasts. sEVs derived from normoxic LOXL2 OE Cal-27 cells significantly induced the phosphorylation of FAK and Src in sEV-treated fibroblasts. Consistently, sEVs derived from hypoxic LOXL2 KD Cal-27 cells significantly inhibited FAK/Src phosphorylation in recipient fibroblasts (**Figure S5**A). To investigate the role of FAK/Src signaling in mediating FN production by fibroblasts, MRC5 cells were treated with sEVs derived from wild-type or LOXL2 OE Cal-27 and SCC-9 cells in the presence of either FAK or Src inhibitor. sEVs derived from LOXL2 OE HNSCC cells significantly induced the production of FN by sEV-treated MRC5 cells. The LOXL2-enriched sEV-induced FN production was significantly inhibited by both FAK and Src inhibitors, suggesting that LOXL2-induced FN production depends on the activation of FAK/Src signaling (**Figure S**5B). We then investigated whether FAK/Src signaling mediates LOXL2-induced pre-metastatic niche, sEVs derived from wild-type or LOXL2 OE Cal-27 cells with or without FAK/Src inhibitors were i.v. injected. After 6-week injection, GFP+ Cal-27 cells were i.v. injected and the lung tissue samples were analyzed for tumor colonization as described above. LOXL2-enriched sEV-treated mice showed a significant increase in colonized tumor cells in the lung compared with that in wild-type sEV-treated mice (*P* < 0.001, **Figure S**5C). LOXL2-enriched sEV-induced colonization in mice lung was significantly inhibited by the administration of either FAK or Src inhibitor, which was, to a certain degree, restored by sFN supplementation (**Figure S**5C).

Myeloid-derived suppressor cells (MDSCs) are enriched in pre-metastatic niches; therefore, we evaluated the recruitment of Gr-1 cells in the lungs of sEV-treated mice. Immunofluorescence and flow cytometry data showed that treatment with hypoxic sEVs induced significant infiltration of MDSCs in the lung, which was inhibited by BAPN (**Figure** 5F). The inhibitory effects of BAPN on MDSC infiltration were rescued by the administration of sFN (**Figure** 5F). Consistently, normoxic sEVs with LOXL2 OE induced MDSC infiltration, which was significantly inhibited by both FAK and Src inhibitors. The decreased MDSC infiltration caused by FAK/Src inhibitors was remarkably rescued by sFN treatment (**Figure S**5D). These results suggest that LOXL2-enriched sEVs can prime pre-metastatic niche formation by stimulating FN production in a FAK/Src-dependent manner in fibroblasts.

### Circulating sEV-LOXL2 predicts poor survival

**Figure 6:**
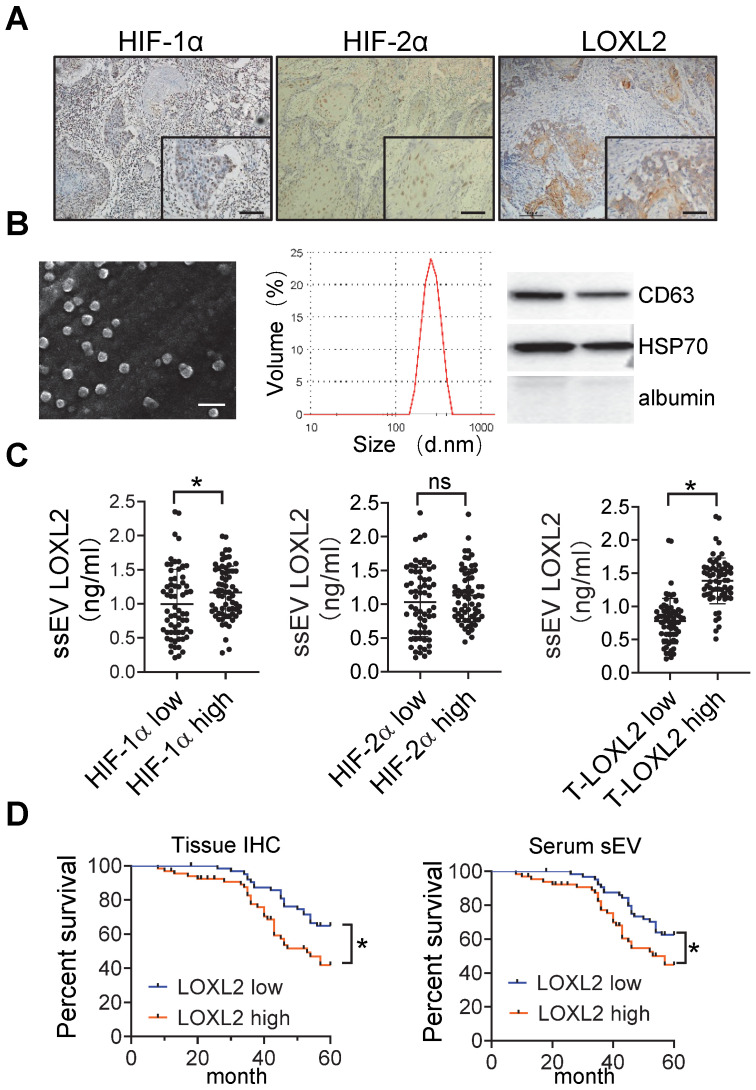
** circulating sEV-LOXL2 predicts poor survival.** (A) Immunohistochemical staining of HIF-1α, HIF-2α, and LOXL2 were performed on HNSCC tissues. Bar: 50 μm; (B) sEVs derived from serum were validated by SEM (left), DLS (middle), and Western blotting (right panel); (C) LOXL2 expression in serum sEVs were measured by ELISA. n=130, *P <* 0.05, ns: non-significant; (D) Kaplan-Meier survival curve for overall survival according to IHC staining of LOXL2 in OSCC tissues (left panel) and LOXL2 levels in serum sEVs (right panel). n=130 patients in both groups, * *P* < 0.05

Immunohistochemical staining of HIF-1α, HIF-2α, and LOXL2 was performed in the HNSCC tissue samples (**Figure 6**A). The percentage of positive cells was estimated using an image analysis system, and the median values were regarded as the cutoff values for low and high expression. We isolated sEVs from the serum of patients with HNSCC. The size distribution of the purified serum sEVs (ssEVs) was consistent (50-300 nm) according to the results of SEM and DLS (**Figure** 6B). Western blotting analysis showed that these sEVs are enriched with CD63 and HSP70 but not with albumin (**Figure** 6B).

LOXL2 expression in serum sEVs was measured by ELISA. HNSCC tissues with high HIF-1α but not HIF-2α levels corresponded to a significant increase in the level of LOXL2 in the serum sEVs (*P* = 0.032, **Figure** 6C). In addition, serum sEV-LOXL2 levels correlated with the expression of LOXL2 in the HNSCC tissues (*P* < 0.001, **Figure** 6C right panel).

The overall survival of patients was analyzed based on LOXL2 levels within the serum sEVs and tissue expression of LOXL2. LOXL2 expression levels in the HNSCC tissues (*P* = 0.023) and serum (*P* = 0.006) sEVs were significantly correlated with poor overall survival of patients with HNSCC (**Figure** 6D).

In addition to tissue and ssEV LOXL2, HIF-1α and HIF-2α expression, sex, age, T stage, lymph node involvement, and histologic differentiation were included in the univariate and multivariate analyses performed by Kaplan-Meier survival analysis and Cox proportional hazard regression model respectively. The univariate analyses showed that patients with high HIF-1α expression (*P <* 0.001, **Figure S6**A), high HIF-2α expression (*P =* 0.005, **Figure S**6B), advanced T stage (*P =* 0.002, **Figure S**6C), lymph node involvement (*P <* 0.001, **Figure S**6D), or poor histologic differentiation (*P =* 0.001, **Figure S**6E) had significantly decreased 5-year overall survival (**Table 1**). No significant differences were observed in age (*P* = 0.7, **Figure S**6F) or sex (*P* = 0.11, **Figure S**6G) in the overall survival analyses. To investigate whether ssEV LOXL2 could be an alternative to tissue LOXL2 as an independent prognostic indicator for overall survival, tissue LOXL2 or ssEV LOXL2 were included in the multivariate analyses using a forward variable selection technique. In the univariate analysis with tissue LOXL2, HIF-1α (HR: 4.79, 95% CI: 2.41-9.49, *P <* 0.001), tissue LOXL2 (HR: 2.99, 95% CI: 1.65-5.41, *P* < 0.001), lymph node involvement (HR: 2.33, 95% CI: 1.11-4.9, *P* = 0.02), and histologic differentiation (poor vs. well, HR: 2.6, 95% CI: 1.08-6.46, *P* = 0.03) were remained in the regression model (**Table** 1). In the Cox proportional hazard regression analysis with ssEV LOXL2, HIF-1α (HR: 3.48, 95% CI: 1.83-6.61, *P* < 0.001), lymph node involvement (HR: 2.66, 95% CI: 1.28-5.6, *P =* 0.009), and ssEV LOXL2 (HR: 2.26, 95% CI: 1.29-3.9, *P =* 0.004) remained significant in the model (**Table 1**). These results suggest that serum sEV LOXL2 can reflect a hypoxic and aggressive tumor type and that serum sEV-LOXL2 levels could serve as an alternative to tissue LOXL2 as an independent prognostic factor of overall survival for patients with HNSCC.

## Discussion

The “seed and soil” theory described by Stephen Paget 35 in the 1880s implies the role of the tumor microenvironment (TME) in the initiation and maintenance of tumorigenesis. Hypoxia is one of the most important characteristics of TME, and is defined as a reduction in the normal level of tissue oxygen tension 7, 36. The hypoxic TME is involved in many “hallmarks of cancer” 37, such as angiogenesis, reprogramming energy metabolism, evading immune destruction, activating invasion and metastasis, tumor-promoting inflammation, sustaining proliferative signaling, resisting cell death, and genome instability. Here, we demonstrated that hypoxia contributes to HNSCC cell invasion, and to the formation of pre-metastatic niches via secretion of sEVs. These hypoxic cell-derived sEVs can deliver increased levels of LOXL2 to non-hypoxic HNSCC cells to elicit an EMT phenotype and induce invasion. Importantly, LOXL2-enriched sEVs can be incorporated by distant fibroblasts inducing an increased production of FN, which recruits additional MDSCs to form a pre-metastatic niche for disseminated HNSCC cells. These results collectively suggest that the local hypoxic tumor microenvironment encodes metastatic information in sEVs, which regulate multiple steps of metastasis, including local invasion and remote adhesion.

An imbalance between O_2_ demand and supply within tumors leads to progressively lower O_2_ levels as the distance from the vessels increases; most parts of solid tumors are hypoxic compared to surrounding normal tissue 38, 39. Tumor cells respond to reduced O_2_ supply by changes in gene expression that are mainly mediated by O_2_-regulated HIFs 40. Hypoxia inhibits prolyl hydroxylation, ubiquitination, and proteasomal degradation of HIF-α, resulting in the stabilization and rapid accumulation of HIF-α and transcription of hundreds of downstream target genes 40. HIF transcript genes have pleiotropic effects on local aspects of cancer pathobiology, including angiogenesis, metabolic reprogramming, ECM remodeling, EMT, motility, invasion, metastasis, stem cell maintenance, and immune evasion 38. These local effects of hypoxia have been extensively studied in the past decades; however, the remote function of hypoxia is poorly understood.

Although being insufficiently investigated, hypoxia has been suggested to stimulate the formation of PMN because PMN-stimulating cytokines and growth factors secreted by the primary tumor, such as VEGF, PGF, tumor necrosis factor-α, and transforming growth factor-β (TGF-β) are substantially enhanced by hypoxia 6. These tumor-secreted factors are accompanied by the recruitment of various bone marrow-derived cell (BMDC) populations, which further contribute to PMN and aid cancer cell extravasation 4. These primary tumor-secreted proteins are presumed to be diluted in the systemic circulation and randomly disseminated in the body. However, the organ distribution of metastases is not random. Thus novel mechanisms, in addition to primary tumor-secreted cytokines/chemokines, may induce the formation of PMNs in an organ-specific manner. Recently, tumor cell-derived sEVs, carrying tumor-specific molecules were suggested to mediate the remote tumor-host interactions and contribute to the formation of organ-specific PMNs.

Tumor-derived sEVs, which are absorbed by organ-specific cells, have been shown to establish the PNN 41. Proteomic analyses of sEVs revealed distinct integrin expression patterns in which integrin α_6_β_4_ and α_6_β_1_-containing sEVs were associated with lung metastasis, while integrin α_v_β_5_-containing sEVs were linked to liver metastasis 41. Targeting integrin α_6_β_4_ and α_v_β_5_ decreased sEV uptake and lowered lung and liver metastasis, respectively 41. Moreover, macrophage migration inhibitory factor (MIF) was found to be highly expressed in pancreatic ductal adenocarcinoma cell-derived sEVs, and its blockade prevented liver PMN formation and metastasis 42. In addition to proteins, sEV-miR-25-3p was found to be involved in PMN formation by promoting vascular permeability and angiogenesis in colorectal cancer 43. Melanoma cell-derived sEVs were shown to induce lung PMN formation by delivering small nucleic RNAs, which activate toll-like receptor 3 in lung epithelial cells 44. The present study is the first to demonstrate that HNSCC-derived sEVs contribute to PMN formation by stimulating FN production by fibroblasts. Overall, the roles of sEVs in PMN formation are diverse and depend on cancer types and range from metabolic reprogramming to recruitment of numerous immune and non-immune stromal cells to facilitate metastatic outgrowth 45.

Our data and the data of several other groups have demonstrated that hypoxia stimulates the production of sEVs and substantially alters the proteomic and nucleic acid profiles of sEVs 11, 14, 46. sEVs mediate a broad range of bidirectional signal transduction processes between various cell types (cancer cell-cancer cell, cancer cell-stromal cell, and stromal cell-stromal cell) within the hypoxic tumor microenvironment and play essential roles in tumor invasiveness, angiogenesis, proliferation, chemotherapy and radiation resistance, immune evasion, metabolism, and cancer stemness 15. Several findings support the involvement of hypoxia in PMN formation; however, evidence of the direct regulation of sEV-mediated PMN formation by hypoxia is limited. Recently, Deep et al.^18^ demonstrated that sEVs derived from hypoxic prostate cancer cells enhanced the levels of MMP2, MMP9, and extracellular matrix proteins (FN and collagen) and increased the number of CD11b+ cells at putative PMN sites. Proteomic profiling performed by liquid chromatography/mass spectrometry identified differentially expressed protein cargos in normoxic and hypoxic sEVs 18. However, it is not clear which sEV-cargo contributes to hypoxic sEV-regulated PMN formation. Here, we provide direct evidence showing that hypoxic HNSCC cell-derived sEVs can deliver LOXL2 to prime the PMN formation by stimulating the FAK/Src signaling-dependent FN production by fibroblasts, which recruits additional MDSCs to the PMN. To the best of our knowledge, this is the first study to show a direct evidence of the effects of tumor cell-derived sEVs on PMN formation in HNSCC.

LOXL2 is a target of HIF 47, 48 and a member of the LOX family of ECM-modifying enzymes comprising LOX and LOXL1-4 that catalyze the crosslinking of collagens and elastin 49. LOXL2-regulated ECM-modification plays important roles in induction of EMT 19, vasculogenic mimicry 50, cell adhesion, senescence, and invasion in the local tumor microenvironment 51. In addition to local function, soluble LOXL2 has been suggested to potentially regulate the PMN formation 47, 52. Direct evidence on LOXL2-induced PMN formation was provided by pioneer work by Cui and colleagues, who demonstrated that matrix stiffness-upregulated LOXL2 promoted recruitment of bone marrow derived cells to assist pre-metastatic niche formation of hepatocellular carcinoma 53. They further showed that tumor cell-secreted LOXL2 stimulated fibronectin and MMP2 expression by lung fibroblasts in a PI3K-AKT dependent manner, by means of which LOXL2 contributes to lung PMS formation and occurrence of lung metastasis of hepatocellular carcinoma 54. Here, we show that LOXL2 delivered by tumor cell-derived sEVs to fibroblasts induced PMN formation of HNSCC. Our results support the conclusion of Cui's work 53, 54, because tumor cell-secreted LOXL2 protein includes soluble form and those encapsulated in extracellular vesicles. Since soluble LOXL2 functions extracellularly while EV-encapsulated LOXL2 functions intracellularly, it is reasonable to hypothesize that soluble and EV-encapsulated LOXL2 may play different roles with different molecular mechanisms. In the regulation of PMN formation, whether soluble or EV-encapsulated LOXL2 plays a leading role remains unknown and represents an interesting topic for further investigation. Another recent study has reported that hypoxic microvascular endothelial cell-derived sEVs regulate focal ECM remodeling by delivering LOXL2 55. In general, the tumor microenvironment contains high number of tumor cells and various stromal cells including microvascular endothelial cells. Therefore, tumor-promoting LOXL2 can be delivered by sEVs that are derived from the tumor and stromal cells in hypoxic tumor microenvironment.

A solid tumor contains well-oxygenated (normoxic), lower O_2_ (hypoxia), and asphyxiated (necrotic) regions 38. Intratumoral hypoxia drives changes in gene expression, leading to alterations in cell signaling that result in cancer progression. Our results showed that hypoxic HNSCC cell-derived sEVs might be distributed to normoxic regions thereby driving non-hypoxic cells toward a pro-metastatic phenotype. These results indicate that hypoxia functions not only on cells within the hypoxic zone but also on cells in the well-oxygenated areas via tumor cell-derived sEVs. Aga et al. 56 found that endogenous HIF-1α is detectable in nasopharyngeal carcinoma cell-derived sEVs. Endogenous HIF-1α retains its DNA-binding activity and is transcriptionally active in recipient cells resulting in reciprocal changes in the expression of E-cadherin and N-cadherin associated with EMT and invasiveness. However, HIF-1α was not detected in normoxic or hypoxic sEVs derived from HNSCC cells in our study. Differences between different types of cancer may contribute to this discrepancy because several other cargos, such as RAB22A, MMP-13, and long non-coding RNA-UCA1, have been demonstrated to contribute to hypoxic sEV-mediated recipient cell progression of various cancer types 21, 57, 58.

In conclusion, we provided direct evidence that the hypoxic tumor microenvironment of HNSCC can drive local invasion of non-hypoxic HNSCC cells and stimulate remote PMN formation. These processes are mediated by HNSCC cell-derived sEVs that deliver LOXL2 to non-hypoxic HNSCC cells and fibroblasts to induce EMT and FN production respectively. However, several main limitations remain in this study. Firstly, sEVs include exosomes and microvesicles which have different biogenesis process but share highly similar composition, size, and functions. Currently, most of the literatures have disregarded the different origins of exosome and microvesicles, it is unfeasible to identify which one is really counted, which is also applicable to the present study. Secondly, sEVs are packed with a variety of cellular components including mRNAs, miRNAs, proteins, and metabolites responsible for different pathophysiological functions. We focused on the protein carried by sEV in present study and cannot rule out the potential contribution of other components, which needs further investigation.

## Supplementary Material

Supplementary figures and table.

## Figures and Tables

**Table 1:** Univariate and multivariable analyses of OS in patients with HNSCC

Univariate		Mean OS (95% CI)	*P*
tissue LOXL2	Low	53.89 (51.6-56.1)	**0.023**
	High	48.1 (44.5-51.6)	
ssEV LOXL2	Low	54.2 (51.9-56.5)	**0.006**
	High	47.7 (44.2-51.2)	
HIF-1α	Low	55.5 (52.8-58.1)	**<0.001**
	High	46.7 (43.6-49.7)	
HIF-2α	Low	53.5 (50.6-56.3)	**0.005**
	High	48.6 (45.4-51.8)	
age	<60y	52.1 (49.5-54.8)	0.7
	≥60y	49.8 (46.3-53.3)	
sex	Female	51.7 (48.4-54.9)	0.1
	Male	50.4 (47.6-53.2)	
T stage	T1/T2	55.6 (52.9-58.2)	**0.002**
	T3/T4	48.3 (45.4-51.3)	
N stage	N0	55.3 (52.2-58.5)	**<0.001**
	N+	48.8 (46-51.6)	
differentiation	well	52.6 (50-55.3)	**0.001**
	moderate	49.6 (45.8-53.5)	
	poor	42 (33.7-50.3)	
**Multivariable with tissue LOXL2**		HR (95% CI)	*P*
HIF-1α	High	4.79 (2.41-9.49)	**<0.001**
tissue LOXL2	High	2.99 (1.65-5.41)	**<0.001**
N stage	N+	2.33 (1.11-4.9)	**0.02**
differentiation	moderate vs. well	0.89 (0.47-1.66)	0.71
	Poor vs. well	2.6 (1.08-6.46)	**0.03**
**Multivariable with ssEV LOXL2**		HR (95% CI)	*P*
HIF-1α	High	3.48 (1.83-6.61)	**<0.001**
N stage	N+	2.66 (1.28-5.6)	**0.009**
ssEV LOXL2	High	2.26 (1.29-3.9)	**0.004**

## Abbreviations

sEV: small extracellular vesicles
HNSCC: head and neck squamous cell carcinoma
LOXL2: lysyl oxidase like 2
EMT: Epithelial-to-mesenchymal transition
PMN: pre-metastatic niche
FN: fibronectin
MDSC: myeloid-derived suppressor cell
HIF: hypoxia-inducible factor
ChIP: chromatin immunoprecipitation
CM: conditioned media
SEM: scanning electron microscopy
DLS: Dynamic light scattering
KD: knockdown
iTRAQ: Isobaric Tagging Technology for Relative Quantitation
KEGG: Kyoto Encyclopedia of Genes and Genomes
GO: Gene Ontology
DEP: differentially expressed protein
ECM: extracellular matrix
TME: tumor microenvironment
BMDC: bone marrow-derived cell
MIF: migration inhibitory factor
qRT-PCR: Quantitative real-time PCR
IF: Immunofluorescence
IHC: immunohistochemistry
