# Supplementary material for: LOXL2-enriched small extracellular vesicles mediate hypoxia-induced premetastatic niche and indicates poor outcome of head and neck cancer
Source: Theranostics. 2021 Sep 3;11(19):9198–216. doi: 10.7150/thno.62455 (PMC8490529; doi:10.7150/thno.62455)
Supplement: Supplementary file 1 — Supplementary figures and table. [file thnov11p9198s1.pdf]

Table s1 significantly differentially expressed proteins between normoxic and hypoxic sEVs

Gene name	Cal27_Nor	Cal27_Nor	Cal27_Nor	Cal27_Hyp	Cal27_Hyp	Cal27_Hyp	mean Nor	mean Hyp	Hyp/Nor Ratio	Hyp/Nor *P* value
LOXL2	0.410	0.390	0.420	0.964	1.125	1.158	0.407	1.082	2.661	0.000
COL4A2	0.751	0.669	0.798	1.927	1.828	1.679	0.739	1.811	2.450	0.000
SDC4	0.612	0.559	0.621	1.183	1.189	1.235	0.597	1.202	2.013	0.000
F10	0.488	0.469	0.552	0.711	0.783	0.892	0.503	0.795	1.581	0.007
SERPINE1	0.851	0.785	0.914	1.333	1.239	1.351	0.850	1.308	1.538	0.001
LDHA	0.889	0.882	0.793	1.295	1.285	1.336	0.855	1.305	1.527	0.000
LAMB2	1.049	1.069	1.115	1.565	1.625	1.513	1.078	1.568	1.455	0.000
NOTCH3	0.973	0.873	0.886	1.384	1.236	1.258	0.911	1.293	1.419	0.002
ANXA2	1.042	1.062	1.158	1.581	1.527	1.446	1.087	1.518	1.396	0.001
GAPDH	1.186	1.187	1.058	1.575	1.628	1.559	1.144	1.587	1.388	0.001
LAMB2	0.981	0.885	0.956	1.239	1.289	1.236	0.941	1.255	1.334	0.001
PLOD1	0.784	0.669	0.778	0.988	0.932	1.025	0.744	0.982	1.320	0.007
PGM1	0.917	0.879	0.992	1.066	1.169	1.368	0.929	1.201	1.292	0.045
EEF2	1.174	1.082	1.043	1.410	1.442	1.398	1.100	1.417	1.288	0.002
SFPQ	0.898	0.792	0.892	1.082	1.028	1.177	0.861	1.096	1.273	0.013
APOB	0.853	0.832	0.849	1.004	1.054	1.156	0.845	1.071	1.268	0.007
C1R	1.394	1.256	1.146	1.523	1.552	1.628	1.265	1.568	1.239	0.018
PXDN	1.554	1.254	1.368	1.812	1.752	1.532	1.392	1.699	1.220	0.066
STC2	1.046	1.058	1.063	1.213	1.235	1.358	1.056	1.269	1.202	0.009
FN1	1.124	1.158	1.028	1.255	1.256	1.336	1.103	1.282	1.162	0.019
PKM	1.048	0.963	1.158	1.177	1.217	1.256	1.056	1.217	1.152	0.058
COL4A1	0.997	0.965	0.889	1.096	1.125	1.058	0.950	1.093	1.150	0.019
WDR1	1.096	1.088	0.998	1.156	1.165	1.258	1.061	1.193	1.125	0.043
FLNA	1.090	1.125	1.102	0.984	0.962	0.952	1.106	0.966	0.874	0.001
COL5A1	1.644	1.789	1.755	1.515	1.598	1.356	1.729	1.490	0.861	0.045
C3	1.880	1.899	1.962	1.731	1.468	1.736	1.914	1.645	0.860	0.043
FLNB	1.263	1.363	1.351	1.089	1.036	0.998	1.326	1.041	0.785	0.002
LGALS3BP	1.465	1.558	1.685	1.284	1.236	1.165	1.569	1.228	0.783	0.009
BMP1	1.290	1.280	1.360	1.045	1.022	0.998	1.310	1.022	0.780	0.001
DCN	2.113	2.236	2.336	1.744	1.785	1.663	2.228	1.731	0.777	0.003
SNRPD2	1.137	1.169	1.256	0.928	0.932	0.856	1.187	0.905	0.762	0.003
COL7A1	0.901	0.995	1.023	0.740	0.785	0.698	0.973	0.741	0.762	0.007
CFH	1.610	1.630	1.580	1.169	1.250	1.190	1.607	1.203	0.749	0.000
FBN2	1.448	1.489	1.526	1.195	1.095	1.036	1.488	1.109	0.745	0.002
PAPPA	1.056	1.125	1.089	0.721	0.825	0.882	1.090	0.809	0.743	0.005
FBLN1	1.915	1.925	1.995	1.369	1.439	1.522	1.945	1.443	0.742	0.001
PCOLCE2	1.012	1.025	1.158	0.779	0.885	0.698	1.065	0.787	0.739	0.018
B2M	1.929	1.956	1.885	1.459	1.426	1.369	1.923	1.418	0.737	0.000
TCP1	1.219	1.258	1.248	0.919	0.885	0.826	1.242	0.877	0.706	0.000
PSMA7	1.537	1.489	1.589	1.067	1.120	1.056	1.538	1.081	0.703	0.000
CHGB	1.231	1.150	1.320	0.908	0.820	0.770	1.234	0.833	0.675	0.003
MYL6	1.398	1.269	1.289	0.972	0.889	0.756	1.319	0.872	0.662	0.004
COL2A1	1.156	1.256	1.169	0.731	0.842	0.732	1.194	0.768	0.644	0.001
EEF1A1P5	1.290	1.330	1.210	0.955	0.885	0.621	1.277	0.820	0.643	0.013
COL12A1	2.141	2.028	2.258	1.326	1.269	1.362	2.142	1.319	0.616	0.000
CCT3	1.379	1.362	1.256	0.850	0.940	0.630	1.332	0.807	0.605	0.006
RPS16	1.004	1.032	0.889	0.754	0.559	0.426	0.975	0.580	0.595	0.020
COL1A1	1.154	1.158	1.036	0.673	0.587	0.639	1.116	0.633	0.567	0.001
EFEMP1	1.800	1.898	1.969	1.039	1.125	0.956	1.889	1.040	0.551	0.000
THBS1	2.298	2.136	2.058	1.167	1.025	1.084	2.164	1.092	0.505	0.000
TFPI	1.103	1.025	1.125	0.658	0.521	0.425	1.084	0.535	0.493	0.002
COL1A2	1.046	1.039	1.136	0.531	0.521	0.523	1.074	0.525	0.489	0.000
FLNB	1.393	1.332	1.258	0.850	0.620	0.460	1.328	0.643	0.485	0.005
CTGF	1.982	1.952	1.881	0.849	0.789	0.962	1.938	0.867	0.447	0.000
COCH	0.568	0.559	0.652	0.288	0.211	0.185	0.593	0.228	0.384	0.001

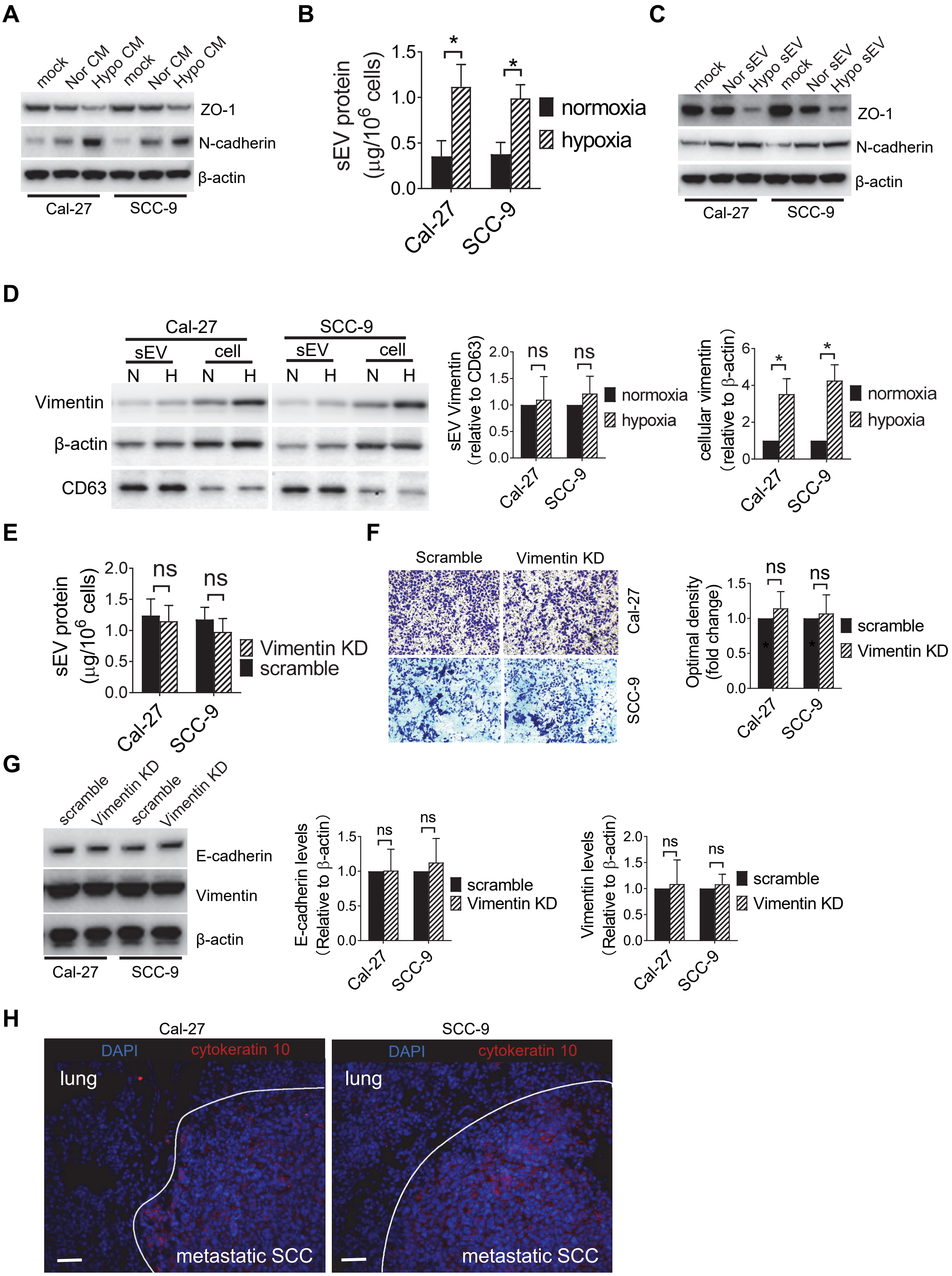

A
mock
Nor CM
Hypo CM
ZO-1
N-cadherin
β-actin
Cal-27
SCC-9
B
sEV protein (μg/10^6 cells)
normoxia
hypoxia
Cal-27
SCC-9
C
mock
Nor sEV
Hypo sEV
ZO-1
N-cadherin
β-actin
D
sEV
cell
N
H
Vimentin
β-actin
CD63
ns
sEV Vimentin (relative to CD63)
cellular vimentin (relative to β-actin)
E
sEV protein (μg/10^6 cells)
Vimentin KD
scramble
F
Scramble
Vimentin KD
Optimal density (fold change)
G
E-cadherin
Vimentin
E-cadherin levels (Relative to β-actin)
Vimentin levels (Relative to β-actin)
H
DAPI
cytokeratin 10
lung
metastatic SCC

Figure s1

(A)Normoxic and hypoxic CM-treated Cal-27 and SCC-9 cells were measured for ZO-1 and N-cadherin expression by and Western blotting. (B) The amount of sEVs was determined by the protein concentration as determined by BCA assay. Data represent at least three experiments performed in triplicate. * $P < 0.05$; (C) Normoxic and hypoxic sEV-treated Cal-27 and SCC-9 cells were measured for ZO-1 and N-cadherin expression by Western blotting. (D) E-cadherin and vimentin expression in Cal-27 and SCC-9 cells and their sEVs were measured by Western blotting (left panel). Cellular E-cadherin and vimentin expression were quantified relative to β-actin (right panel). E-cadherin and vimentin expression in sEVs was quantified relative to CD63 (middle panel). Data represent at least three experiments performed in triplicate. * $P < 0.05$, ns: non-significant. (E) The amount of sEVs was determined by the protein concentration determined by BCA assay. Data represent at least three experiments performed in triplicate. ns: non-significant; (F) Invasion assays were performed with Cal-27 and SCC-9 cells seed on 12μm insets which were placed on 24-well plates supplemented with sEVs derived from Cal-27 and SCC-9 cells respectively. Cells that migrated to the bottom surface were stained with crystal violet and observed by light microscopy (lower panel, magnification, 200). Quantitative analysis of crystal violet optical density (right panel). Data represent at least three experiments performed in triplicate.; ns: non-significant; (G) E-cadherin and vimentin expression levels in Cal-27 and SCC-9 cells treated with wildtype or vimentin KD sEVs were measured by Western blotting (left panel). Quantification of Western blots was performed relative to β-actin (right panel). Data represent at least three experiments performed in triplicate. ns: non-significant. (H) The metastatic tumors were detected by IF staining of cytokeratin 10.

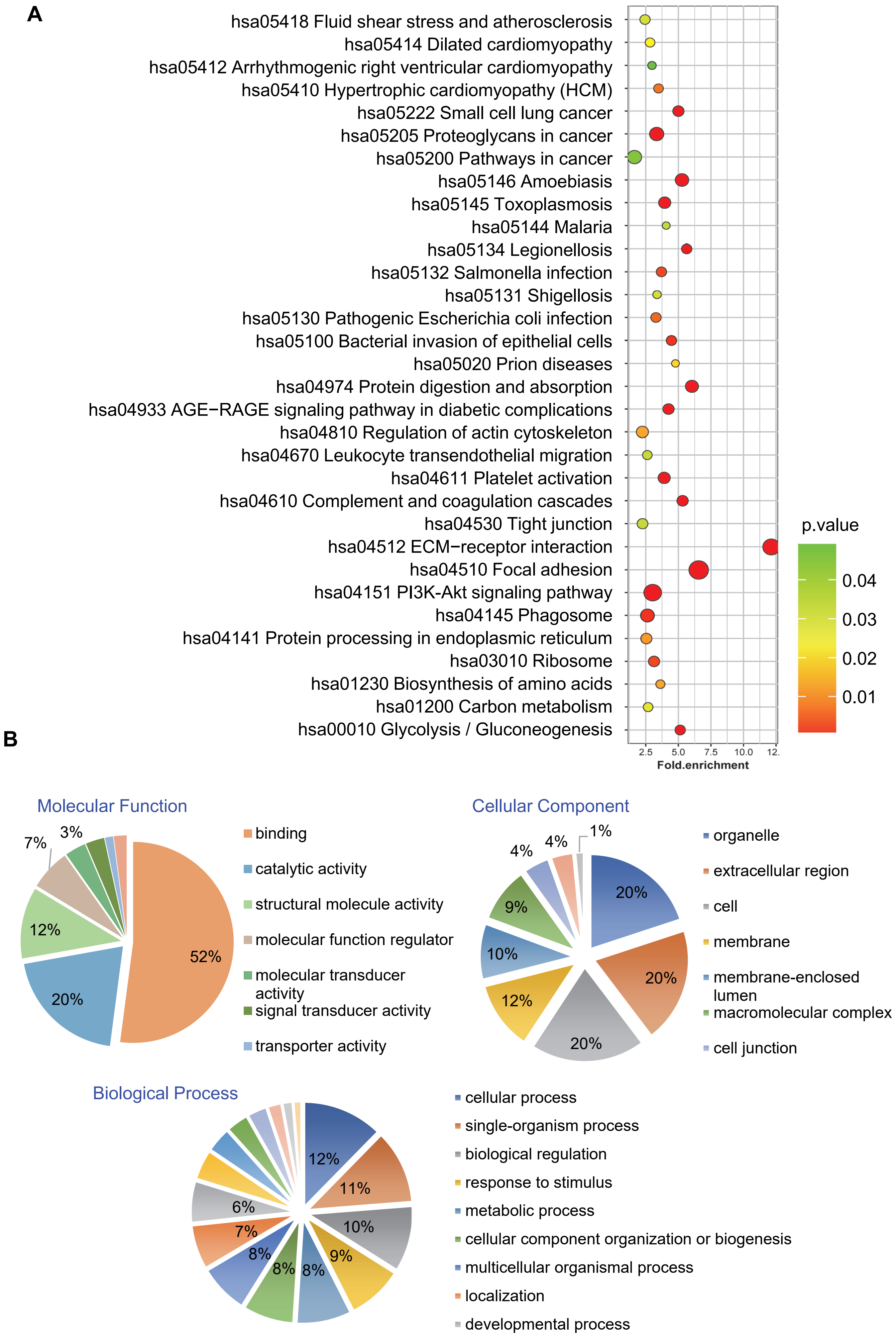

A
hsa05418 Fluid shear stress and atherosclerosis
hsa05414 Dilated cardiomyopathy
hsa05412 Arrhythmogenic right ventricular cardiomyopathy
hsa05410 Hypertrophic cardiomyopathy (HCM)
hsa05222 Small cell lung cancer
hsa05205 Proteoglycans in cancer
hsa05200 Pathways in cancer
hsa05146 Amoebiasis
hsa05145 Toxoplasmosis
hsa05144 Malaria
hsa05134 Legionellosis
hsa05132 Salmonella infection
hsa05131 Shigellosis
hsa05130 Pathogenic Escherichia coli infection
hsa05100 Bacterial invasion of epithelial cells
hsa05020 Prion diseases
hsa04974 Protein digestion and absorption
hsa04933 AGE−RAGE signaling pathway in diabetic complications
hsa04810 Regulation of actin cytoskeleton
hsa04670 Leukocyte transendothelial migration
hsa04611 Platelet activation
hsa04610 Complement and coagulation cascades
hsa04530 Tight junction
hsa04512 ECM−receptor interaction
hsa04510 Focal adhesion
hsa04151 PI3K-Akt signaling pathway
hsa04145 Phagosome
hsa04141 Protein processing in endoplasmic reticulum
hsa03010 Ribosome
hsa01230 Biosynthesis of amino acids
hsa01200 Carbon metabolism
hsa00010 Glycolysis / Gluconeogenesis
2.5
5.0
7.5
10.0
12.
Fold.enrichment
p.value
0.04
0.03
0.02
0.01
B
Molecular Function
52%
20%
12%
7%
3%
binding
catalytic activity
structural molecule activity
molecular function regulator
molecular transducer activity
signal transducer activity
transporter activity
Cellular Component
20%
20%
20%
12%
10%
9%
4%
4%
1%
organelle
extracellular region
cell
membrane
membrane-enclosed lumen
macromolecular complex
cell junction
Biological Process
12%
11%
10%
9%
8%
8%
8%
7%
6%
cellular process
single-organism process
biological regulation
response to stimulus
metabolic process
cellular component organization or biogenesis
multicellular organismal process
localization
developmental process

Figure s2

(A)Identified protein pathways were annotated by KEGG, which showed that ECM-receptor interaction was among the most important pathways. (B) The identified proteins were further classified by GO annotation into three categories: molecular function (upper left), cellular compartment (upper right), and biological process (lower).

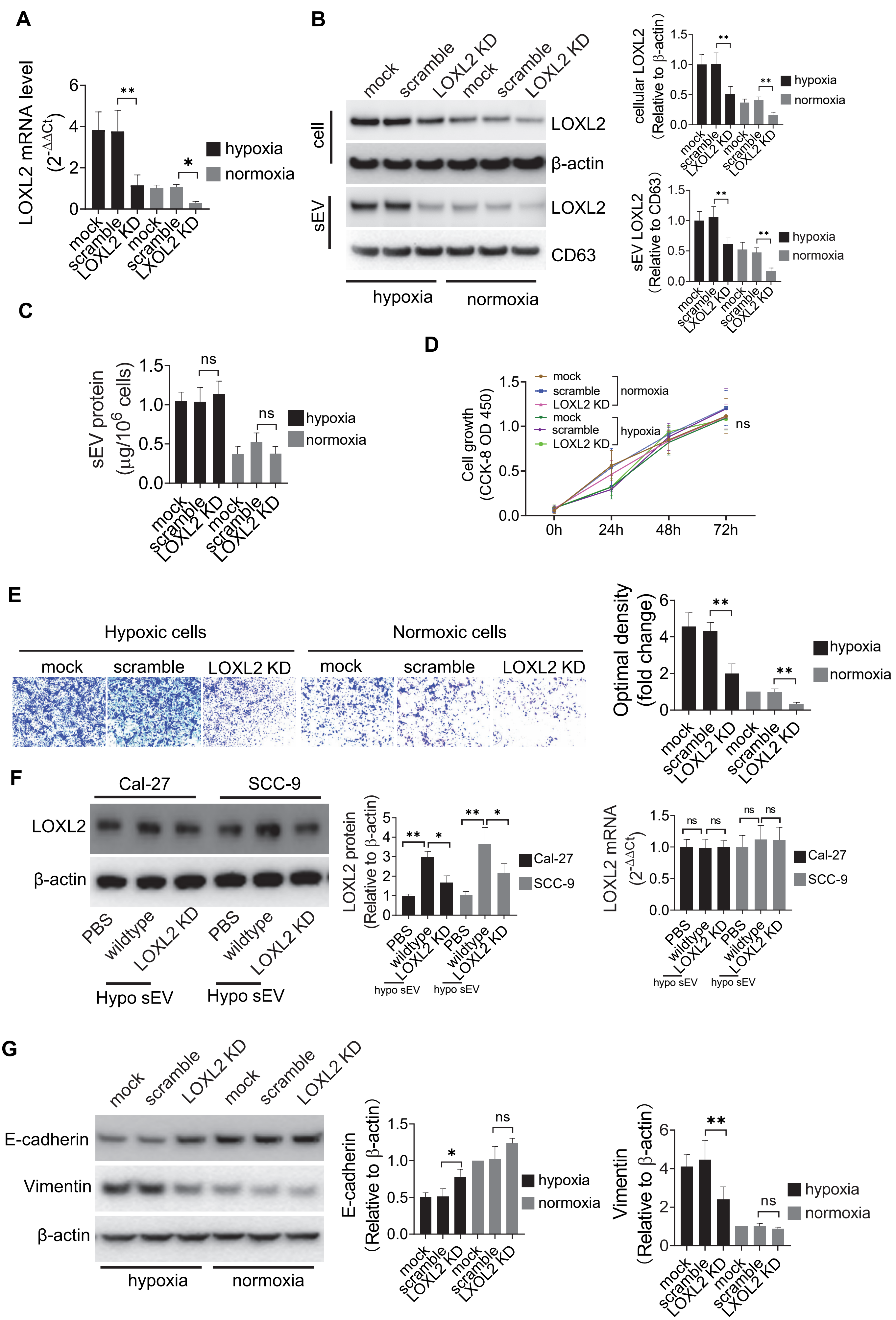
A
LOXL2 mRNA level
(2-ΔΔCt)
6
4
2
0
**
*
hypoxia
normoxia
mock
scramble
LOXL2 KD
mock
scramble
LXOL2 KD
B
mock
scramble
LOXL2 KD
mock
scramble
LOXL2 KD
cell
sEV
LOXL2
β-actin
LOXL2
CD63
hypoxia
normoxia
cellular LOXL2
(Relative to β-actin)
1.5
1.0
0.5
0.0
**
**
hypoxia
normoxia
sEV LOXL2
(Relative to CD63)
1.5
1.0
0.5
0.0
**
**
hypoxia
normoxia
mock
scramble
LXOL2 KD
mock
scramble
LOXL2 KD
C
sEV protein
(μg/10^6 cells)
1.5
1.0
0.5
0.0
ns
ns
hypoxia
normoxia
mock
scramble
LOXL2 KD
mock
scramble
LOXL2 KD
D
Cell growth
(CCK-8 OD 450)
1.5
1.0
0.5
0.0
0h
24h
48h
72h
mock
scramble
LOXL2 KD
normoxia
mock
scramble
LOXL2 KD
hypoxia
ns
E
Hypoxic cells
Normoxic cells
mock
scramble
LOXL2 KD
mock
scramble
LOXL2 KD
Optimal density
(fold change)
6
4
2
0
**
**
hypoxia
normoxia
mock
scramble
LOXL2 KD
mock
scramble
LOXL2 KD
F
Cal-27
SCC-9
LOXL2
β-actin
PBS
wildtype
LOXL2 KD
PBS
wildtype
LOXL2 KD
Hypo sEV
Hypo sEV
LOXL2 protein
(Relative to β-actin)
5
4
3
2
1
0
**
*
**
*
Cal-27
SCC-9
PBS
wildtype
LOXL2 KD
PBS
wildtype
LOXL2 KD
hypo sEV
hypo sEV
LOXL2 mRNA
(2-ΔΔCt)
1.5
1.0
0.5
0.0
ns
ns
ns
ns
Cal-27
SCC-9
PBS
wildtype
LOXL2 KD
PBS
wildtype
LOXL2 KD
hypo sEV
hypo sEV
G
mock
scramble
LOXL2 KD
mock
scramble
LOXL2 KD
E-cadherin
Vimentin
β-actin
hypoxia
normoxia
E-cadherin
(Relative to β-actin)
1.5
1.0
0.5
0.0
*
ns
hypoxia
normoxia
mock
scramble
LOXL2 KD
mock
scramble
LXOL2 KD
Vimentin
(Relative to β-actin)
6
4
2
0
**
ns
hypoxia
normoxia
mock
scramble
LOXL2 KD
mock
scramble
LXOL2 KD

Figure s3

(A) The mRNA levels in LOXL2 shRNA transfected cells were evaluated by qRT-PCR. Data represent at least three experiments performed in triplicate. * $P < 0.05$, ** $P < 0.001$; (B) LOXL2 protein expression levels in cells and sEVs were measured by Western blotting (left panel). Cellular LOXL2 protein levels were quantified relative to β-actin (upper right panel). sEVs LOXL2 protein levels were quantified relative to CD63 (lower right panel). Data represent at least three experiments performed in triplicate. * $P < 0.001$; (C) sEV production was quantified by sEV protein concentration as determined by BCA assays. Data represent at least three experiments performed in triplicate. ns: non-significant; (D) Cell growth was measured by CCK-8 assays. Data represent at least three experiments performed in triplicate. ns: non-significant; (E) Role of LOXL2 KD on cell migration was evaluated by Transwell assays. Data represent at least three experiments performed in triplicate. ** $P < 0.001$; (F) Cal-27 and SCC-9 cells were treated by sEVs which were derived from wildtype and LOXL2 KD cells respectively. LOXL2 protein levels in sEV recipient cells were measured by Western blotting (left panel). Quantification of Western blots were performed relative to β-actin (middle panel). LOXL2 mRNA levels in sEV-treated cells were measured by qRT-PCR (right panel). Data represent at least three experiments performed in triplicate. * $P < 0.05$, ** $P < 0.001$, ns: non-significant

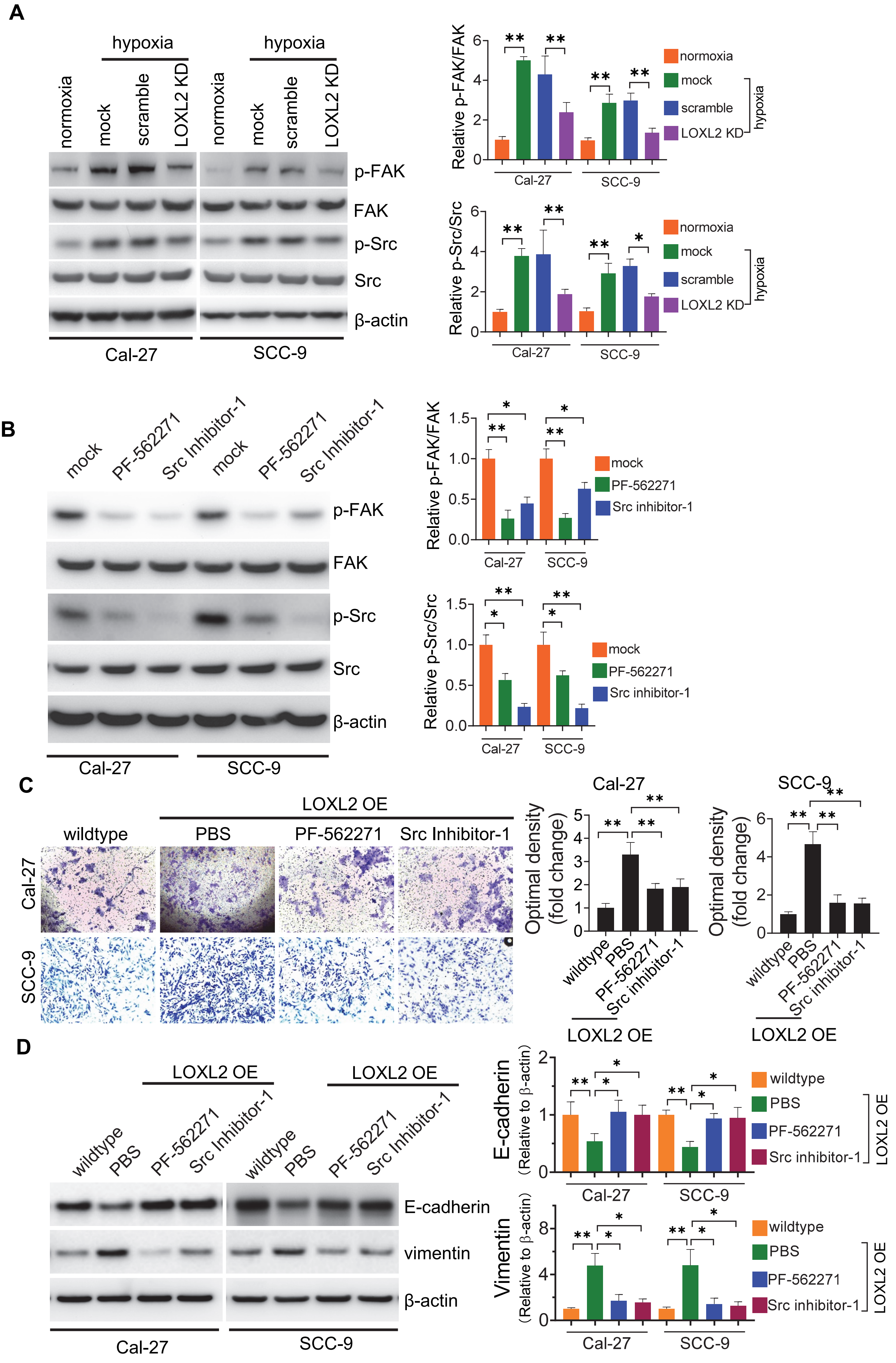
A
hypoxia
hypoxia
normoxia
mock
scramble
LOXL2 KD
p-FAK
FAK
p-Src
Src
β-actin
Cal-27
SCC-9
Relative p-FAK/FAK
Relative p-Src/Src
normoxia
mock
scramble
LOXL2 KD
hypoxia
B
mock
PF-562271
Src Inhibitor-1
Relative p-FAK/FAK
Relative p-Src/Src
Src inhibitor-1
C
LOXL2 OE
wildtype
PBS
Optimal density (fold change)
D
E-cadherin
vimentin
E-cadherin (Relative to β-actin)
Vimentin (Relative to β-actin)

Figure s4

(A) The levels of p-FAK and p-Src in normoxic and hypoxic cells with or without LOXL2 KD were measured by Western blotting (left panel). Quantification of Western blots was performed. Data represent at least three experiments performed in triplicate. * $P < 0.05$, ** $P < 0.001$; (B) p-FAK and p-Src levels in hypoxic Cal-27 and SCC-9 cells were evaluated by Western blotting. Quantification of Western blots was performed (right panel). Data represent at least three experiments performed in triplicate. * $P < 0.05$, ** $P < 0.001$; (C) Cal-27 and SCC-9 cell invasion were measured by invasion assays. Cells that migrated to the bottom surface were stained with crystal violet and observed by light microscopy (magnification, 200) (left panel). Quantitative analysis of crystal violet optical density (right panel). Data represent at least three experiments performed in triplicate. ** $P < 0.001$; (D) E-cadherin and vimentin protein levels were measured by Western blotting (left panel). Quantification of Western blots was performed. Data represent at least three experiments performed in triplicate. * $P < 0.05$, ** $P < 0.001$

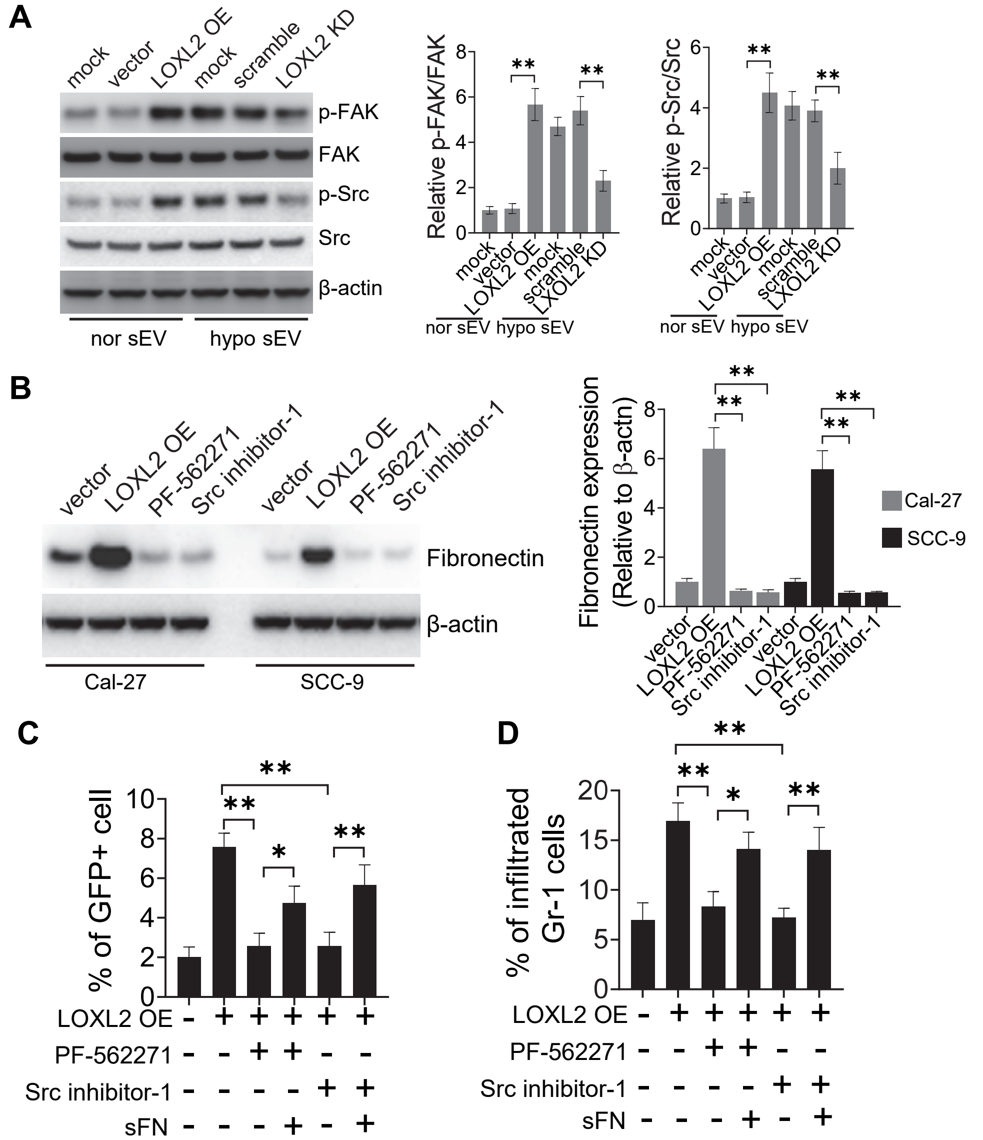

Figure s5

(A) LOXL2 OE Cal-27 cells were cultured under normoxic condition; LOXL2 KD Cal-27 cells were cultured under hypoxic condition. Equal amount of sEVs obtained from these cells were added to MRC5 fibroblasts. The phosphorylation of FAK and Src in the sEV-treated fibroblasts were measured by Western blotting (left panel). Quantification of Western blots was performed. Data represent at least three experiments performed in triplicate. ***P* < 0.001; (B) MRC5 cells were treated with sEVs derived from wildtype or LOXL2 OE Cal-27 and SCC-9 cells at the presence of either FAK or Src inhibitor. The FN levels were measured by Western blotting (left panel). Quantification of Western blots was performed. Data represent at least three experiments performed in triplicate. ***P* < 0.001; (C) sEVs derived from wildtype or LOXL2 OE Cal-27 cells with or without FAK/Src inhibitors were i.v. injected to nude mice twice a week. After 6-week injection, GFP+ tumor cells were i.v. injected. One week later, mice were euthanized and the GFP+ cells were quantified by flow cytometry n=6, ** *P*<0.001; (D) Gr-1+ myeloid cell infiltration in the sEV-treated lungs was measured by flow cytometry. n=6, * *P* < 0.05, ***P*<0.001.

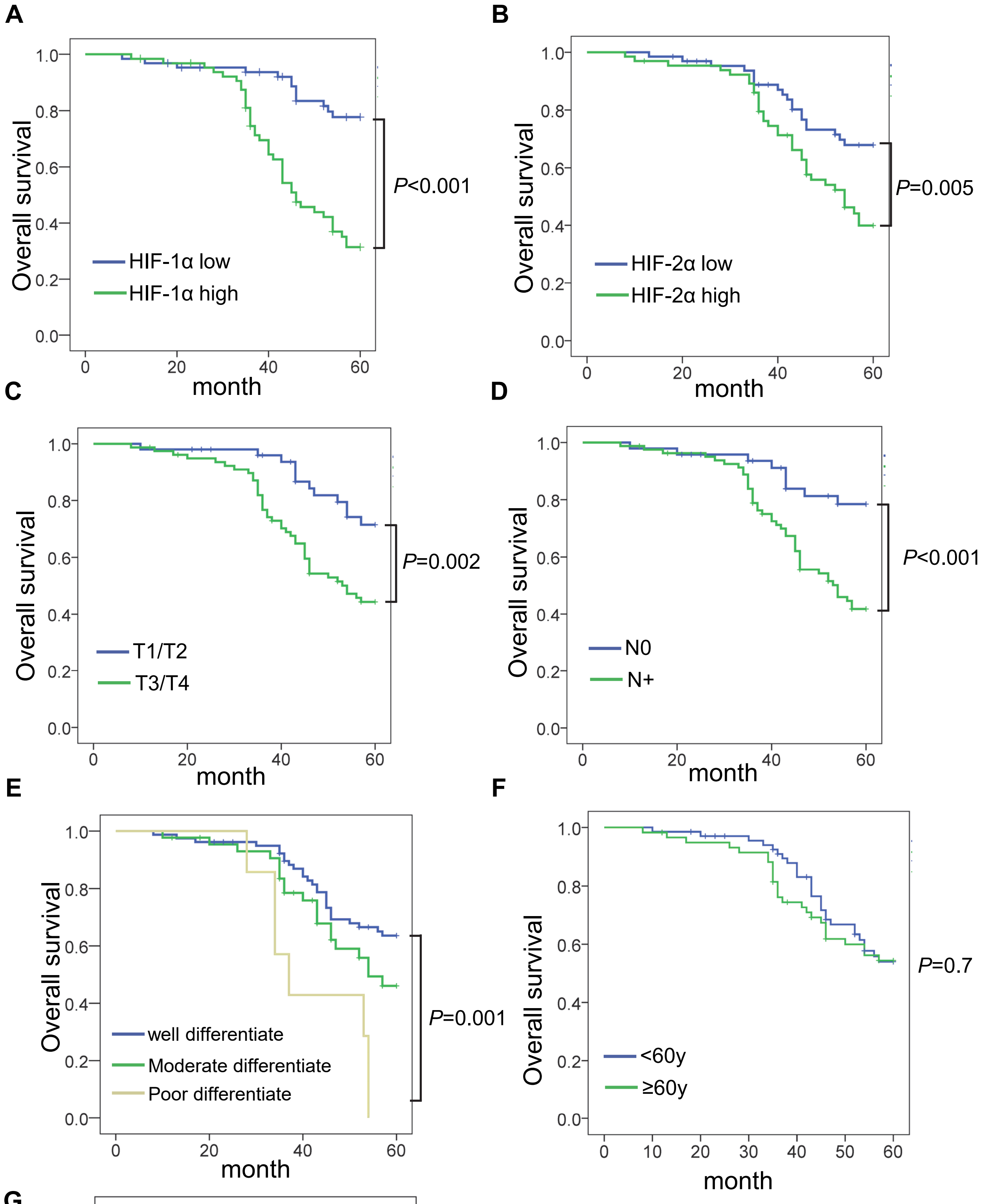

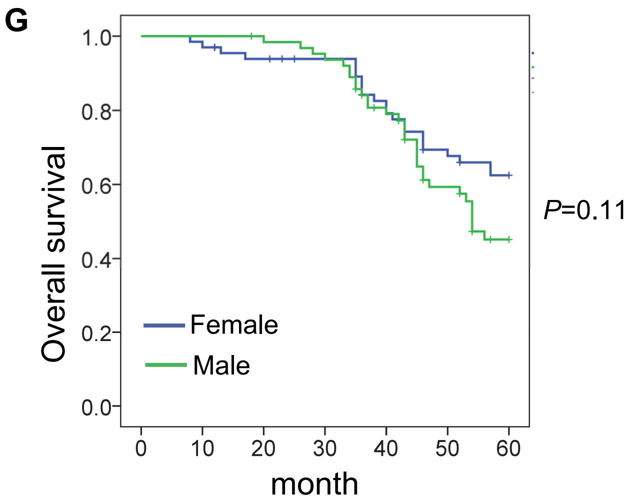

Figure s6

Kaplan-Meier survival curve for overall survival according to HIF-1α staining (A), HIF-2α staining (B), T stage (C), lymph node involvement (D), histologic differentiation (E), age (F), and sex (G). n=130
